# Supplementary material for: Finding shortest and nearly shortest path nodes in large substantially incomplete networks by hyperbolic mapping
Source: Nat Commun. 2023 Jan 17;14:186. doi: 10.1038/s41467-022-35181-w (PMC9845360; doi:10.1038/s41467-022-35181-w)
Supplement: Supplementary file 1 — Supplementary Information [file 41467_2022_35181_MOESM1_ESM.pdf]

SUPPLEMENTARY INFORMATION: Finding shortest and nearly shortest path nodes in large substantially incomplete networks by hyperbolic mapping

Maksim Kitsak,[1,2] Alexander A. Ganin,[3,4] Ahmed Elmokashfi,[5] Hongzhu Cui,[6,7]
Daniel A. Eisenberg,[8] David L. Alderson,[9] Dmitry Korkin,[6,10,11] and Igor Linkov[12]

[1]Faculty of Electrical Engineering, Mathematics and Computer Science, 2600 GA Delft, The Netherlands
[2]Network Science Institute, Northeastern University, 177 Huntington avenue, Boston, MA, 022115
[3]University of Virginia, Department of Systems and Information Engineering, Charlottesville, VA, 22904, USA
[4]U.S. Army Engineer Research and Development Center, Contractor, Concord, MA, 01742, USA
[5]Simula Research Lab, Oslo, Norway
[6]Bioinformatics and Computational Biology Program,
Worcester Polytechnic Institute, Worcester, MA 01609, USA
[7]Institute for Genomic Medicine, Columbia University Medical Center, New York, NY, USA
[8]Operations Research Department, Naval Postgraduate School, Monterey, CA, 93943, USA
[9]Department of Operations Research, Naval Postgraduate School, Monterey, CA, 93943, USA
[10]Computer Science Department, Worcester Polytechnic Institute, Worcester, MA 01609, USA
[11]Data Science Program, Worcester Polytechnic Institute, Worcester, MA 01609, USA
[12]U.S. Army Engineer Research and Development Center,
Environmental Laboratory, Concord, MA, 01742, USA
(Dated: November 24, 2022)

Contents

I. Why the Shortest Path Problem is Hard in Incomplete Networks 2
A. The shortest path problem 2
B. Real networks are substantially incomplete 2

II. Hyperbolic Space 3
A. Hyperboloid model 3
B. Beltrami-Klein disk model 3
C. Visualizing hyperbolic geodesics 4
D. Distance to hyperbolic geodesic 5
E. Fitting hyperbolic geodesic 6

III. Random Hyperbolic Graphs 7

IV. Learning Hyperbolic Representations of Incomplete Real Networks 8
A. Why hyperbolic embeddings are robust to *uniformly* missing links 9

V. The Internet at the Autonomous System Level and BGP Routing 10
A. Internet as a network of Autonomous Systems 10
B. Internet data 11
C. Mapping Internet networks 11
D. Interdomain routing and the border gateway protocol 11
E. Internet communication security and prefix hijacks 12
F. Prefix hijack data 13
G. Existing methods to detect prefix hijacks 13
H. Hyperbolic stretch as a measure of path conformity 14

VI. Protein-Protein Interaction Network and Cellular Pathways 15
A. Protein interaction data 15
B. Similarity-based representation of protein interactions 15
C. Mapping the similarity-based protein interaction network 16
D. Latent-geometric alignment and genetic composition of cellular pathways 17
E. Genes in the latent-geometric vicinity of the UPP pathway 17

VII. Pretty-Good-Privacy (PGP) web of trust network 20

VIII. Identification of Nearly Shortest Path Nodes: Definitions and Metrics 20
A. Network-based and random-walk based path finding metrics. 21
B. Path finding on synthetic networks. 21
C. Path finding on incomplete networks: the similarity-based PPI network and the PGP web of trust 22
D. Path finding on networks with spurious links. 22

References 25

I. WHY THE SHORTEST PATH PROBLEM IS HARD IN INCOMPLETE NETWORKS

A. The shortest path problem

The shortest path problem in incomplete networks is extra hard as it combines two non-trivial problems. The first problem is the shortest path problem in a fully observed network. The second problem is completing the network by identifying missing nodes and links.

There are a plethora of graph-theoretic methods for finding shortest paths, provided the network of interest is fully known. The most famous methods, to this end, are versions of the Dijkstra and Bellman-Ford algorithms [1]. These methods guarantee the exact solution to the problem in a polynomial time, as long as the entire network is known. To find shortest paths, graph-theoretic methods are based on the exploration of network topology. The Dijkstra algorithm, for instance, proceeds by finding paths from the origin node to its immediate neighbors first and then neighbors of neighbors, proceeding iteratively until it reaches the destination node [2]. Since Dijkstra, along with other graph-theoretic algorithms, explore network topology, missing or spurious network links and nodes will inevitably result in incorrect inference. This is the case since even an addition or deletion of a single link may completely change the shortest path set. A clear example here is adding a direct link connecting the origin-destination node pair. In this case, the newly added link will create a direct shortest path, making all previous shortest paths obsolete.

Existing methods to reconstruct missing data in networks are primarily focused on finding missing links [3, 4]. Despite recent advances in missing link prediction methods, combining missing link and path finding methods into a single framework is infeasible. Indeed, most missing link inference methods only provide a ranking of missing links. Ranking scores allow one to identify the most likely *individual* missing link candidates but cannot quantify the joint likelihood that several links are missing simultaneously. While some missing link identification methods, e.g., network embedding methods, explicitly compute probabilities for missing link candidates, examining all possible combinations of missing links that may result in the shortest path is a highly non-trivial problem that has not been solved.

There is a family of works that aim at finding shortest paths in weighted networks with uncertain weights [5–14]. These works, however, assume that all network links are known, which greatly reduces the space of possible shortest paths connecting the pair of nodes of interest.

B. Real networks are substantially incomplete

Despite our best efforts at collecting large datasets, our knowledge of many crucial networked systems remains incomplete.

Communication security. The lack of global knowledge of the Internet's topology can be exploited to compromise Internet interdomain routing, leading to either loss of communication data or rerouting data flows through undesired entities [15–27]. The global Internet topology is not readily known due to its distributed nature and privacy considerations. The changes in the interdomain Internet topology occur on a time scale of minutes [28].

Epidemic spreading. To control the spreading of contagious diseases, such as COVID-19, we need accurate epidemic forecast and contact tracing tools that can operate on substantially incomplete and dynamic human mobility data. The coverage of digital contact tracing tools is extremely limited, accounting from 1% to 20% of population in different countries [29].

Cellular pathways and Biomedicine. We need methods to map unknown parts of cellular pathways and quantify the effects of genetic mutations on cellular pathways in the context of substantially incomplete networks of molecular interactions. Despite the sustained recent progress of protein-protein interaction measurement techniques, the number of missing human protein-protein interactions exceeds that of experimentally documented interactions [30].

II. HYPERBOLIC SPACE

In mathematics, a homogeneous space that has constant negative curvature is often referred to as a hyperbolic space. Notable models of a hyperbolic space include the hyperboloid, the Beltrami-Klein disk, the Poincaré disk, the Hemisphere and the Poincaré half-plane models [31]. In our work we primarily use the 2-dimensional hyperboloid model, with the exception of drawing hyperbolic geodesics, where we utilize the 2-dimensional Beltrami-Klein disk model.

A. Hyperboloid model

We refer to the 2-dimensional hyperboloid model as $\mathbb{H}^2$. To define $\mathbb{H}^2$ of curvature $K = -\zeta^2 < 0$ we consider the upper sheet of the 2-dimensional hyperboloid:

$$x_0^2 - x_1^2 - x_2^2 = \frac{1}{\zeta^2} = -\frac{1}{K},\ x_0 > 0 \tag{1}$$

in the 3-dimensional Minkowski space with metric:

$$\mathrm{d}s^2 = -\mathrm{d}x_0^2 + \mathrm{d}x_1^2 + \mathrm{d}x_2^2. \tag{2}$$

The polar coordinate representation on the hyperboloid (r, θ) is defined by

$$\begin{aligned} x_0 =& \frac{1}{\zeta}\cosh \zeta r, \\ x_1 =& \frac{1}{\zeta}\sinh \zeta r \cos\theta, \\ x_2 =& \frac{1}{\zeta}\sinh \zeta r \sin\theta, \end{aligned} \tag{3}$$

where $r > 0$ and $\theta \in [0, 2\pi]$ are the radial and angular coordinates, respectively.

The coordinate transformation in (3) yields the $\mathbb{H}^2$ metric

$$\mathrm{d}s^2 = \mathrm{d}r^2 + \frac{1}{\zeta^2}\sinh^2(\zeta r)\,\mathrm{d}\theta^2. \tag{4}$$

The distance d_{ij} between two points i and j in $\mathbb{H}^2$ is given by the hyperbolic law of cosines:

$$\cosh \zeta d_{ij} = \cosh \zeta r_i \cosh \zeta r_j - \sinh \zeta r_i \sinh \zeta r_j \cos \Delta\theta_{ij}, \tag{5}$$

where $\Delta\theta_{ij}$ is the angle between i and j:

$$\Delta\theta_{ij} = \pi - |\pi - |\theta_i - \theta_j||. \tag{6}$$

For sufficiently large ζr_i and ζr_j values, and $\Delta\theta_{ij} > 2\sqrt{e^{-2\zeta r_i} + e^{-2\zeta r_j}}$, the hyperbolic law of cosines in Eq. (5) is closely approximated by

$$d_{ij} = r_i + r_j + \frac{2}{\zeta}\ln\left(\sin(\Delta\theta_{ij}/2)\right). \tag{7}$$

Since changing the curvature parameter ζ is equivalent to the rescaling of radial coordinates, without loss of generality, throughout this work we set ζ to

$$\zeta = 1. \tag{8}$$

B. Beltrami-Klein disk model

The hyperboloid model $\mathbb{H}^2$ is closely related to the Beltrami-Klein disk model. The latter can be obtained from the hyperbolic disk model by projecting points of the latter through the origin $(x^0 = x^1 = x^2 = 0)$ onto the plain $x^0 = 1$, see Fig. S1.

An important property of the Beltrami-Klein disk model is that hyperbolic geodesics in it are represented by straight lines. This property allows us to draw hyperbolic geodesics first in the Beltrami-Klein disk and then project them onto the hyperboloid model, see Section II C.

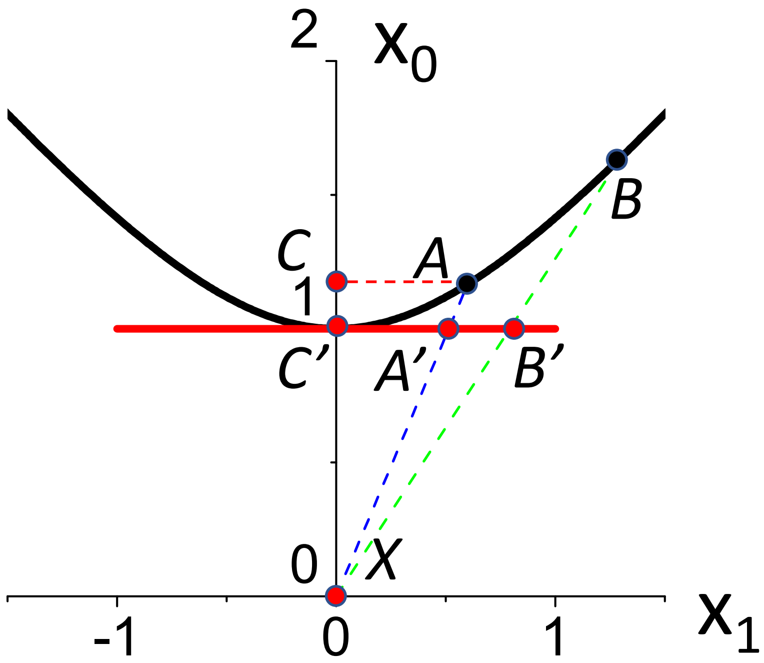

FIG. S1: **Relation between the hyperboloid and the Klein disk models.** Shown is the $x_2 = 0$ slice of the 3-dimensional Minkowski space. Shown in black is the hyperboloid $x_0^2 - x_1^2 - x_2^2 = 1$, and shown in red is the Klein disk $(x_1)^2 + (x_2)^2 \leq 1$, $x_0 = 1$. Point A' and B' on the Klein disk are obtained by projecting points A and B on the hyperboloid through the origin $(x_0 = x_1 = x_2 = 0)$ onto the plain $x_0 = 1$, as shown by dashed lines.

C. Visualizing hyperbolic geodesics

To draw the hyperbolic geodesic $\gamma(A, B)$ between two points A and B, with Cartesian coordinates $(x_{0,A},\ x_{1,A})$ and $(x_{0,B},\ x_{1,B})$ in $\mathbb{H}^2$ we first project these points onto the Klein model [31]. Since $\triangle XAC \sim \triangle XA'C'$ and $C'X = 1$, we have that $r_A^{KL} \equiv C'A' = CA/CX = x_{1,A}/x_{0,A}$. Since A lies on the hyperboloid, $x_{0,A}^2 = 1 + x_{1,A}^2$, and $r_A^{KL} = x_{0,A}/\sqrt{1 + x_{1,A}^2}$. The expression r_B^{KL} can be obtained following the same steps. Finally, using Eq. (3), we obtain

$$r_{\{A,B\}}^{KL} = \tanh\left(r_{\{A,B\}}\right), \tag{9}$$
$$\theta_{\{A,B\}}^{KL} = \theta_{\{A,B\}}, \tag{10}$$

and for Cartesian coordinates

$$x_{\{A,B\}}^{KL} = r_{\{A,B\}}^{KL} \cos\left(\theta_{\{A,B\}}\right), \tag{11}$$
$$y_{\{A,B\}}^{KL} = r_{\{A,B\}}^{KL} \sin\left(\theta_{\{A,B\}}\right), \tag{12}$$

for points A and B

Since hyperbolic geodesics in the Klein model are straight lines we next fit line $y = kx + b$ (or $y = c$, in case $x_A^{KL} = x_B^{KL}$) between points A and B in the Klein model, obtaining

$$k = \frac{y_A^{KL} - y_B^{KL}}{y_A^{KL} - y_B^{KL}}, \tag{13}$$
$$b = \frac{y_A^{KL} x_B^{KL} - y_B^{KL} x_A^{KL}}{x_A^{KL} - x_B^{KL}} \tag{14}$$

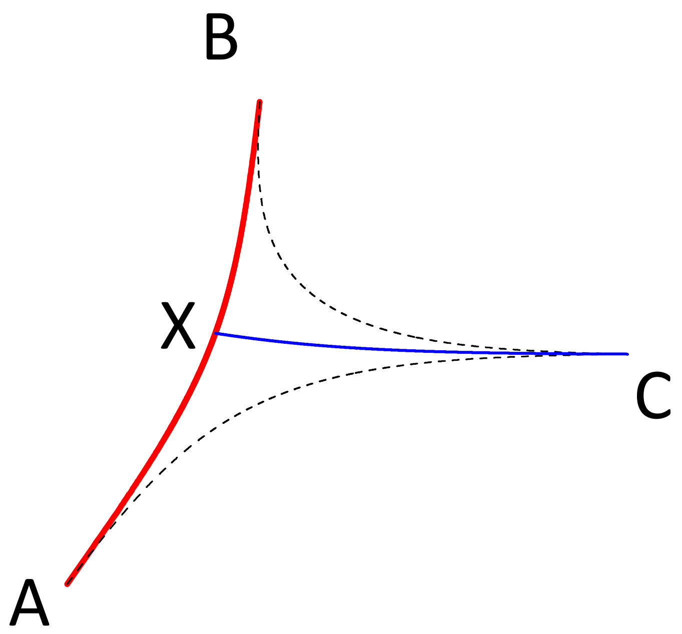

FIG. S2: **Distance to hyperbolic geodesic $\gamma(A,B)$ from point C.** Red solid line denotes the hyperbolic geodesic $\gamma(A,B)$ connecting points A and B. Point X on $\gamma(A,B)$ is closest to point C, and distance $d(C,X)$ is the thought distance from C to $\gamma(A,B)$. The corresponding $\gamma(C,X)$ geodesic is shown with solid blue line. Dashed lines represent geodesics drawn between node pairs A-C and B-C.

Then, the geodesic $\gamma(A,B)$ is constructed as a parametric function in the Klein model:

$$x^{KL}(t) = x_A^{KL} + t\left(x_B^{KL} - x_A^{KL}\right), \quad (15)$$

$$y^{KL}(t) = kx^{KL}(t) + b, \quad (16)$$

$$t \in [0,1], \quad (17)$$

and then $\gamma(A,B)$ is projected back to the $\mathbb{H}^2$ using Eqs. (9) and (10):

$$r(t) = \tanh^{-1}\left[\sqrt{\left(x^{KL}(t)\right)^2 + \left(y^{KL}(t)\right)^2}\right], \quad (18)$$

$$\theta(t) = \tan^{-1}\left[\frac{y^{KL}(t)}{x^{KL}(t)}\right]. \quad (19)$$

D. Distance to hyperbolic geodesic

In this section we derive the exact and approximate expressions for the distance from point C to the hyperbolic geodesic $\gamma(A,B)$ passing points A and B.

Distance to geodesic is defined as the shortest distance from C to point X that belongs to the geodesic:

$$d(C,\gamma(A,B)) \equiv \min \ d(C,X), \quad (20)$$

$$\text{s.t.} \ \ X \in \gamma(A,B) \quad (21)$$

Exact result. Consider the hyperbolic triangle formed $\triangle ACX$ in Fig. S2. According to the hyperbolic law of sines,

$$\frac{\sin(\theta_{AXC})}{\sinh d(A,C)} = \frac{\sin(\theta_{XCA})}{\sinh d(X,A)} = \frac{\sin(\theta_{CAX})}{\sinh d(X,C)}, \quad (22)$$

resulting in

$$\sinh d(X,C) = \frac{\sin(\theta_{CAX})}{\sin(\theta_{AXC})} \sinh d(A,C). \tag{23}$$

If one slides X from A to B along geodesic $\gamma(A,B)$, $\sinh d(A,C)$ and (θ_{CAX}) remain constant and $d(X,C)$ depends only on $\sin(\theta_{AXC})$. Thus, it follows from Eq. (23) that the smallest $d(X,C)$ is achieved when $\sin(\theta_{AXC})$ takes the largest value, i.e., $\theta_{AXC} = \frac{\pi}{2}$. Therefore,

$$\sinh d(X,C) = \sin(\theta_{CAX}) \sinh d(A,C). \tag{24}$$

In other words, similar to the *Euclidean* case, $d(X,C)$ falls onto $\gamma(A,B)$ at the right angle. To determine $d(X,C)$ we need angle θ_{CAX}, which can be found by applying the hyperbolic law of cosines to $\triangle ABC$:

$$\cos\theta_{CAX} = \frac{\cosh d(AB)\cosh d(AC) - \cosh d(B,C)}{\sinh d(AB)\sinh d(AC)}. \tag{25}$$

Taken together, we get

$$d(C,\gamma(A,B)) = \sinh^{-1}\left[\sin(\theta_{CAX})\sinh d(A,C)\right], \tag{26}$$

$$\theta_{CAX} = \cos^{-1}\left[\frac{\cosh d(AB)\cosh d(AC) - \cosh d(B,C)}{\sinh d(AB)\sinh d(AC)}\right]. \tag{27}$$

Approximate result. While the result of Eq. (26) is exact, computing $d(C,\gamma(A,B))$ with it is not straightforward in the case $d(AB)$, $d(AC)$, $d(BC)$ are large. This is the case due to the exponential growth of the hyperbolic functions and the necessity to deal with a combination of extremely large and small numbers to compute θ_{CAX}.

To derive the approximate expression for $d(C,\gamma(A,B))$ we assume that $d(A,X) \gg 1$, $d(B,X) \gg 1$ and $d(C,X) \gg 1$. In this case, we employ the approximate law of hyperbolic cosines, Eq. (7), to approximate $d(A,C)$ and $d(B,C)$ as

$$d(A,C) = d(A,X) + d(C,X) + 2\ln\sin\left(\frac{\theta_{AXC}}{2}\right), \tag{28}$$

$$d(B,C) = d(B,X) + d(C,X) + 2\ln\sin\left(\frac{\theta_{BXC}}{2}\right), \tag{29}$$

and, $d(C,X)$ can be obtained from adding these equations:

$$2d(C,X) = d(A,C) + d(B,C) - d(A,B) - 2\ln\left[\sin\left(\frac{\theta_{AXC}}{2}\right)\sin\left(\frac{\theta_{BXC}}{2}\right)\right] \tag{30}$$

In the derivation of the exact result we established that $\theta_{AXC} = \theta_{BXC} = \frac{\pi}{2}$. Therefore, we obtain from Eq. (30)

$$d(C,\gamma(A,B)) = d(C,X) \approx \frac{1}{2}\left[d(A,C) + d(B,C) - d(A,B)\right] + \ln 2, \tag{31}$$

concluding the derivation.

Finally, the constant additive factor of $\ln 2$ in Eq. (31) is not needed if one's goal is to rank network nodes based on their proximity to the geodesic and can be dropped to simplify the expression.

We emphasize that the relation in Eq. (31) is approximate and is expected to work in cases $d(A,X) \gg 1$, $d(B,X) \gg 1$, and $d(C,X) \gg 1$. These assumptions hold in most practical cases, since the majority of nodes of interest have degrees $k_i \ll N$ and, therefore, are characterized by radial coordinates $r_i \gg 1$.

E. Fitting hyperbolic geodesic

Here we describe our approach to fit a geodesic to a collection of points on $\mathbb{H}^2$. Our approach is analogous to the standard least squares line fit in *Euclidean* space. Given a collection of points $\{C_i\}$, $i = 1, ..., m$, with $\mathbb{H}^2$ coordinates $\{r_i, \theta_i\}$ we are looking to find two points A and B in $\mathbb{H}^2$, such that the geodesic $\gamma(A,B)$ minimizes the sum of the squared distances from points of interest in the $\mathbb{H}^2$:

$$\min \sum_{i=1}^{m} d\left(C_i, \gamma(A,B)\right)^2. \tag{32}$$

When fitting the geodesic we aim to find points with largest possible radial coordinates, $A(r_{\max}, \theta_A)$ and $B(r_{\max}, \theta_B)$, where

$$r_{\max} = \max\{r_i\}, \quad i = 1, ..., m. \tag{33}$$

Then, the problem reduces to finding angular coordinates θ_A and θ_B of the two points, which we find numerically. To this end, we split the unit circle into $\mathcal{O}(N)$ segments, where N is the number of nodes in the network of interest and determine the thought angular coordinates by the brute force.

The rationale behind the brute force search is as follows. The brute force optimization is robust to possible non-convexity of the $\sum_{i=1}^{m} d\left(C_i, \gamma A, B\right)^2$ objective function. Even though the resulting running time complexity of the algorithm, $\mathcal{O}(N^2)$, is rather high, it is on par with that of the HyperLink Embedder, that maps the network of interest to $\mathbb{H}^2$, Section IV. In order to determine angular coordinates of network nodes in $\mathbb{H}^2$, the HyperLink Embedder also discretizes the angular space into $\mathcal{O}(N)$ segments, such that the resulting accuracy of learned angular coordinates is similar to that of the angular coordinates of nodes θ_A and θ_B, describing the geodesic.

III. RANDOM HYPERBOLIC GRAPHS

Path reconstruction in real networks relies on learning their hyperbolic representations. We outline the basic principles of the learning algorithm in Section IV. Here we discuss the Random Hyperbolic Graph (RHG), which serves a null model in this algorithm.

RHGs have been extensively studied in the literature [32–39] and have been shown to reproduce common properties of many real networks including heterogeneous distributions of node degrees, strong clustering, as well as community structure [33, 40, 41].

The latent space of the RHG is the $\mathbb{H}^2$, which we define in Section II A.

The RHG has three parameters — hyperbolic disk radius $R_H > 0$, temperature $T \in [0, 1)$ and node density parameter $\alpha > 1/2$ — and is defined as follows:

1. Draw node coordinates $\{r_i, \theta_i\}$, $i = 1, \ldots, N$ in $\mathbb{H}^2$ from pdfs:

$$\theta_i \leftarrow \rho(\theta) \;=\; 1/(2\pi),\;\; \theta_i \in [0, 2\pi], \tag{34}$$

$$r_i \leftarrow \rho(r) \;=\; \frac{\sinh(\alpha r)}{\cosh(\alpha R) - 1},\;\; r_i \in [0, R] \tag{35}$$

2. Compute distances $\{x_{ij}\}$ between all node pairs in $\mathbb{H}^2$ using Eq. (5).

3. Connect node pairs with probability

$$p\left(x_{ij}\right) = \frac{1}{1 + e^{\frac{x_{ij} - R}{2T}}}. \tag{36}$$

Degree distribution. RHGs are characterized by scale-free degree distributions, $P(k) \sim k^{-\lambda}$, where $\lambda = 2\alpha + 1$. Indeed, the expected degree of a node located at (r, θ) is independent of its angular coordinate θ, $\overline{k}(r, \theta) = \overline{k}(r, 0) = \overline{k}(r)$, and is given by

$$\begin{aligned}\overline{k}(r) \;&=\; (N-1)\int \mathrm{d}r'\rho(r')\int \mathrm{d}\theta'\rho(\theta')p\left[x(r, 0, r', \theta')\right] \\ &\approx\; \frac{4N\alpha}{2\alpha - 1}\frac{T}{\sin \pi T}e^{-r/2},\end{aligned} \tag{37}$$

see [33]. The average degree of the model is given by

$$\overline{k} = \int \mathrm{d}r\rho(r)\overline{k}(r) = \frac{8N\alpha^2}{(2\alpha - 1)^2}\frac{T}{\sin \pi T}e^{-R/2}. \tag{38}$$

As seen from Eq. (38), $\overline{k}$ in the most general case depends on the network size N.

To achieve sparse models with $\overline{k}$ independent of N one sets the radius of the hyperbolic disk to

$$R(N) = 2\ln\left(N/\nu\right), \tag{39}$$

where $\nu > 0$ is the tuning parameter, directly related to $\bar{k}$. Indeed, with $R(N)$ given by (39)

$$\bar{k} = \frac{8\nu\alpha^2}{(2\alpha-1)^2}\frac{T}{\sin\pi T}, \tag{40}$$

prescribing the value of ν for the target values of $\bar{k}$, α and T.

It has been shown in [42] that in the sparse limit the probability of a node located at (r,θ) to have k connections can be approximated with the Poisson distribution with the mean of $\bar{k}(r)$:

$$P(k|r) = e^{-\bar{k}(r)}\frac{\left[\bar{k}(r)\right]^k}{k!}. \tag{41}$$

Then degree distribution of the RHG is

$$P(k) = \int \mathrm{d}r\rho(r)P(k|r) \sim k^{-\lambda}, \tag{42}$$

$$\lambda = 2\alpha + 1. \tag{43}$$

It follows from Eqs. (37) and (40) that model parameters α and R can be used to control degree distribution exponent λ and the average degree of the model, respectively.

Degree-dependent clustering coefficient. As seen from Eq. (36), connection probability $p(x)$ decreases exponentially for distances $x > R$ with the rate of $\frac{1}{2T}$. Thus, the temperature parameter T, tunes the role of large distances in the formation of links: the higher the T the more likely are long-distance connections. As a result, T controls the clustering coefficient of the RHG. In the $T \to 0$ limit connections are only possible at hyperbolic distances $x < R$ and the clustering coefficient is maximized. Conversely, the clustering coefficient decreases as T increases and vanishes asymptotically in the $T \geq 1$ case [33].

In summary, the main parameters of the RHG controlling properties of the resulting network models are node density parameter $\alpha > \frac{1}{2}$ and temperature parameter $T \in (0,1)$. Parameter α allows the direct control over the exponent of scale-free degree distribution, since $\lambda = 2\alpha + 1$. Temperature parameter controls the *geometricity* of resulting RHGs since larger T values allow for longer distance connections.

The notable property of the $\mathbb{H}^2$, explaining the success of RHGs in reproducing structural properties of real networks is the exponential expansion of the volume of hyperbolic disks drawn on the hyperboloid. Indeed, the $\mathbb{H}^2$ metric, given by Eq. (4), results in the volume element in $\mathbb{H}^2$:

$$\mathrm{d}V = \frac{1}{\zeta}\sinh\zeta r \mathrm{d}r\mathrm{d}\theta, \tag{44}$$

indicating that the volume of a hyperbolic disk of radius R_H is

$$V(R_H) \propto \cosh\zeta R_H - 1 \sim e^{\zeta R_H}, \tag{45}$$

for $R_H \gg 1$. This is in sharp contrast with *Euclidean* spaces, where volume of a d-dimensional ball grows polynomially as a function of its radius, $V_E \sim R_E^d$, for any finite d. The exponential growth of the volume in the $\mathbb{H}^2$ makes the model similar to trees: the latter require exponential amount of space for branching and can be embedded nearly isometrically into hyperbolic spaces [43]. Many real networks, on the other hand, are characterized by scale-free degree distributions, $P(k) = l(k)k^{-\lambda}$, where $l(k)$ is a slowly varying function, often implying the existence of a hidden tree-like hierarchy [44].

IV. LEARNING HYPERBOLIC REPRESENTATIONS OF INCOMPLETE REAL NETWORKS

In this section we formulate the problem of learning the hyperbolic representation of a network, which is also known as the hyperbolic embedding problem. In this work we do not develop a new embedding algorithm. Instead, we utilize the HyperLink embedder developed by us in a previous work [45]. For network-specific details on embedding the AS-level Internet and the PPI network see Sections V and VI, respectively.

To infer hyperbolic node coordinates of a network of interest with randomly removed links we aim to find the set of node coordinates $\{\mathbf{x}_i\} \equiv \{(r_i,\theta_i)\}$, $i = 1, 2.., N$ maximizing the posterior probability $\mathcal{L}(\{\mathbf{x}_i\}|a_{ij},\mathcal{P},q)$ that node coordinates take particular value in the case the network is generated as the RHG with subsequent random link removal process with rate $1-q$. Here a_{ij} is the network's observed adjacency matrix, and $\mathcal{P}$ is the set of parameters of the RHG, $\mathcal{P} = \{\alpha, T, R\}$.

By the Bayes' rule the thought probability is given by

$$\mathcal{L}\left(\{\mathbf{x}_i\}|a_{ij},\mathcal{P},q\right)=\frac{\mathcal{L}\left(a_{ij}|\{\mathbf{x}_i\},\mathcal{P},q\right)\mathrm{Prob}(\mathbf{x}_i)}{\mathcal{L}\left(a_{ij}|\mathcal{P},q\right)}, \tag{46}$$

where $\mathcal{L}\left(a_{ij}|\{\mathbf{x}_i\},\mathcal{P},q\right)$ is the likelihood that network a_{ij} is generated as RHG with subsequent random link removal, $\mathrm{Prob}(\mathbf{x}_i)$ is the prior probability of node coordinates generated by the RHG, and $\mathcal{L}\left(a_{ij}|\mathcal{P},q\right)$ is the probability that the network has been generated as the RHG with random link removal.

Since node pairs are connected independently, the likelihood is given by

$$\mathcal{L}\left(a_{ij}|\{\mathbf{x}_i\},\mathcal{P},q\right)=\prod_{i<j}\left[\tilde{p}\left(x_{ij}\right)\right]^{a_{ij}}\left[1-\tilde{p}\left(x_{ij}\right)\right]^{1-a_{ij}}, \tag{47}$$

where $\tilde{p}\left(x_{ij}\right)$ is the effective connection probability in the RHG generation process with subsequent random link removal:

$$\tilde{p}\left(x\right)\equiv qp(x), \tag{48}$$

and $p(x)$ is the RHG connection probability function prescribed by Eq. (36). Further, we assume the uniform prior probability

$$\mathrm{Prob}(\mathbf{x}_i)=\frac{1}{(2\pi)^N}\prod_{i=1}^{N}\rho(r_i), \tag{49}$$

where $\rho(r_i)$ are given by Eq. (35).

The HyperLink Embedder aims to find node coordinates $\hat{\mathbf{x}}_i$ maximizing the likelihood $\mathcal{L}\left(\{\mathbf{x}_i\}|a_{ij},\mathcal{P},q\right)$, or equivalently, its logarithm,

$$\ln\mathcal{L}\left(\{\mathbf{x}_i\}|a_{ij},\mathcal{P},q\right)=K+\sum_{i=1}^{N}\ln\rho(r_i)+\sum_{i<j}\left[a_{ij}\ln\tilde{p}\left(x_{ij}\right)+\left(1-a_{ij}\right)\ln\left(1-\tilde{p}\left(x_{ij}\right)\right)\right], \tag{50}$$

where constant K absorbs all terms independent of $\{\mathbf{x}_i\}$.

A. Why hyperbolic embeddings are robust to *uniformly* missing links

The logarithm of the likelihood $\mathcal{L}$ in Eq. (47) can be rewritten as a sum of individual node contributions $\mathcal{L}_i$:

$$\ln\,\mathcal{L}\left(a_{ij}|\{\mathbf{x}_i\},\mathcal{P},q\right)=\frac{1}{2}\sum_i\ln\,\mathcal{L}_i, \tag{51}$$

where

$$\ln\,\mathcal{L}_i=\sum_j a_{ij}\ln\left[\tilde{p}\left(x_{ij}\right)\right]+\sum_j\left(1-a_{ij}\right)\ln\,\left[1-\tilde{p}\left(x_{ij}\right)\right] \tag{52}$$

As seen from Eq. (52), the optimal position of any node i depends not only on positions of its neighbors but also on those of its non-neighbors. Since $\tilde{p}\left(x\right)$ is a decreasing function of distance x, one can think of effective attractions exerted by node's neighbors and effective repulsion of node's non-neighbors. Node's resulting position in the embedding, thus, can be viewed as an equilibrium determined by attractive and repulsive forces. If nodes are distributed uniformly in the embedding space and network links are removed uniformly at random, we expect that the effective forces are still balanced, and the equilibrium does not shift far from its location in the original complete network. Similarly, we expect that latent-geometric geodesics and distances from them to network nodes not to be significantly affected by the missing links.

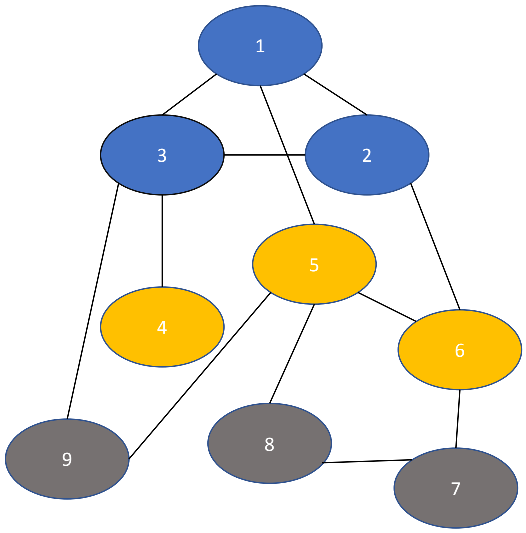

FIG. S3: **A toy AS-level topology.** Nodes are ASes and connections between them represent peering relationships. The blue ASes are tier-1 ISPs, while the orange are regional providers and the grey ones are Enterprise networks and content providers. ASes 1,2,3,5 and 6 are transit providers.

V. THE INTERNET AT THE AUTONOMOUS SYSTEM LEVEL AND BGP ROUTING

A. Internet as a network of Autonomous Systems

At the most basic level, the Internet is a collection of computers (e.g. personal devices, machines and servers) that can exchange data amongst themselves. Each computer is identified with a string of bits called an IP address. A group of adjacent IP addresses are aggregated into a coarser identifier, which is called an IP prefix. A prefix or a group of prefixes are exclusively owned and managed by a single administrative entity known as an autonomous system (AS). Accordingly, the Internet is a network of ASes that interact to facilitate data exchange between their users. ASes differ by their role. Some ASes serve as transit providers, connecting different parts of the Internet and carrying data traffic for other ASes. Examples of transit provider ASes are the CenturyLink, TATA Communications and NTT. Transit provider ASes are typically characterized by a large degree and may be simultaneously present in several geographical areas. Other ASes, focus on providing connectivity and content to end users. These ASes include Internet service providers (ISP)s and content providers. Colloquial examples of ISP ASes are Comcast and Vodafone, while the examples of content provider ASes include Google and Netflix.

The AS-level Internet topology describes how ASes interconnect with each other. It is both scale-free and hierarchical [46, 47]. At the top, there is a dense subgraph of large transit providers, the so-called Tier-1 ISPs, that facilitates connecting different parts of the globe. Then there is a number of regional transit and access providers. At the bottom, there is a large number of Enterprise networks and content providers. Figure S3 depicts a toy AS level topology network. The blue ASes are tier-1 ISPs, while the orange are regional providers and the grey ones depicts Enterprise networks and content providers.

B. Internet data

Collecting an accurate AS-level connectivity network data is of paramount importance to researchers and network engineers. Over the past couple of decades, several algorithms have been developed to infer this topology from BGP data, both RIBs and updates e.g. [48, 49]. In this paper, we used two snapshots of the Internet topology both collected by CAIDA.

The first snapshot of the AS Internet has been collected by CAIDA Archipelago project by June 2009 and consists of $N_1 = 23,748$ ASes and $E_1 = 58,414$ AS relationships. This snapshot has been first analyzed and mapped in Ref. [34]. We use this snapshot in all path reconstruction experiments.

The second AS Internet snapshot has been collected by CAIDA by January 1st, 2019, and is publicly available at CAIDA website [1]. The second snapshot is substantially larger than the first one, reflecting the growth of the Internet over a decade. The second AS Internet snapshot consists of $N_2 = 63,463$ ASes and $E_2 = 318,997$ AS relationships. We need the second snapshot to assess the integrity of BGP routing paths.

C. Mapping Internet networks

To obtain hyperbolic maps of the two AS Internet snapshot we used learning methods described in Section SIV.

Path finding experiments on the complete AS topology. We conducted path finding experiments, not involving missing links on the first snapshot of the AS Internet. To this end, we used the original AS coordinates as inferred in Ref. [34]. To avoid the situation of two ASes having exactly the same coordinates we added a small random noise of $a = 10^{-4}$ rads magnitude to the angular coordinates of Internet ASes:

$$\hat{\theta}_i = \theta_i + aX_i, \tag{53}$$

$$X_i \leftarrow U\left(-\frac{1}{2}, \frac{1}{2}\right), \tag{54}$$

where $i = 1 \dots N$.

Path finding experiments on the AS topology with missing links. We conducted path finding experiments using the first snapshot of the AS Internet. For each experiment we first removed existing links of the network with rate q and then mapped the resulting network to the hyperbolic disk $\mathbb{H}^2$ using the HL embedder algorithm developed by us in Ref. [45]. For the mapping procedure we used $\lambda = 2.1$ and $T = 0.7$ parameters, consistent with Ref. [34]

Integrity of BGP routing paths. The large size of the second snapshot of the AS Internet makes its accurate mapping with the HL embedder prohibitively long. While other existing hyperbolic mapping algorithms may offer faster embedding times, their mapping accuracy has not been rigorously studied. To achieve the embedding of the second snapshot, we used the inferred hyperbolic coordinates of the first AS Internet snapshot as fixed anchors for the second snapshot. The unknown AS coordinates of the second Internet snapshot are then inferred with respect to those of the first AS Internet snapshot. For the mapping procedure we used $\lambda = 2.1$ and $T = 0.7$ parameters, consistent with the other mapping procedures.

D. Interdomain routing and the border gateway protocol

The Internet as a communication network is subject to an increasing number of cyberattacks, aiming at either compromising communication flows or gaining unauthorized access to systems. The decentralized nature of most communication networks, including the Internet, has proven to be both their curse and blessing. On the one hand, the absence of a single authority in a communication network allows for the efficient implementation of communication mechanisms and allows for scalable growth of these networks. On the other hand, the absence of a single authority in a large communication network makes the protection of connectionless communication mechanisms extremely hard, if not impossible.

Our knowledge of the Internet topology is limited. At the macroscopic level, the Internet can be viewed as a network of autonomous systems (AS)es, which interact to facilitate data exchange between their users. ASes can be regarded as independent administrative entities governing the communication of a collection of Internet protocol (IP) addresses. Despite the significant progress in Internet topology measurement techniques [48, 49], our knowledge of

---

[1] https://www.caida.org/data/as-relationships/

the Internet topology remains limited. Due to the large Internet size, the changes in AS-level Internet topology occur on a time scale of minutes [28], making it virtually impossible to capture the exact AS-level connectivity state of the Internet.

Since the information on how to reach devices in another AS is not readily available, this reachability information is disseminated among ASes by the Border Gateway Protocol (BGP) [50]. Each AS maintains its own list of paths to destination IP prefixes and stores it in its routing information base (RIB). RIBs are constructed and updated by means of the BGP update messages. Every time a particular AS updates its RIB, it sends BGP updates reflecting these changes to all of its neighbor ASes. After receiving an update message, AS compares the paths contained in the message to those in its RIB and makes changes in its own RIB, if needed. In the case its RIB is updated, AS sends out BGP update messages to its own neighbor ASes. Every time there is a change in AS-level Internet connectivity, BGP updates are sent out iteratively until every RIB has reached its new equilibrium state and no additional BGP updates are needed.

Concurrently with updating their RIBs, BGP routers perform their main function: route information packets to their respective destination IP addresses. Whenever an information packet arrives at the BGP router, the router uses its RIB to identify the best path for the information packet and forwards it to the next AS on this path.

E. Internet communication security and prefix hijacks

ASes accept routing paths advertised by their neighbors without strict integrity tests. This exposes the entire system to a serious attack vector, commonly known as BGP prefix hijacks. During a BGP prefix hijack, an AS may either claim ownership of addresses that are owned by other ASes or announces that it can provide transit to a specific address or a set of addresses. Such an attack can compromise the affected data flows by either exposing them to malicious actors [51] or simply misrouting them [52]. BGP hijacks are becoming increasingly common [53].

In this work, we consider three different prefix hijack events that are qualitatively different from each other. Out hyperbolic-stretch based detection method successfully flags all three cases as anomalies. In the following, we describe the considered hijacks.

1. *Malaysia Telekom incident.* On June 12th 2015, Malaysia Telekom (ASN4788) announced to its transit provider, Level-3 (AS3549), that it can provide transit to 179,000 prefixes almost a third of the entire routing system. In other words, Malaysia Telekom inserted its ASN in paths leading to these destinations. Level-3 is one of the major hubs in the AS level topology, which resulted in amplifying this hijack. The incident lasted for two hours [54].

2. *PJSC Rostelecom incident* On April 26, 2017 PJSC Rostelecom (ASN12389) claimed ownership to 50 prefixes that are owned by 37 other ASes. ASes that belong to financial services like Visa and Mastercards were over represented among the victim ASes. This hijack lasted for a very short period of time, about 7 minutes. While no motivations were disclosed or identified, the attacks bears several suspicious hallmarks [51].

3. *MainOne Telecom incident.* On November 12th, 2018, traffic to 180 prefixes owned by Google suffered outages and poor performance for over an hour. The affected traffic was rerouted through China Telecom (ASN4809), which dropped it at the great firewall of China, which is a system owned by the Chinese government for filtering and censoring Internet content. China Telecom appeared on the paths to Google because one of its customer ASes, MainOne (ASN37282) hijacked these prefixes and announced them to China Telecom. MainOne blamed the error on misconfigurations [55].

The selected three use cases include both origin hijacks (PJSC and MainOne) as well as a man-in-the-middle attacks, where an AS inserts itself in the path (Malaysia Telecom). Considered hijacks differ in their impact patterns. The hijack by Malaysia Telecom has impacted a large portion of the Internet. The other two cases, PJSC and MainOne, impacted a tiny fraction of prefixes. The success of our approach in flagging all these diverse cases as potential hijacks confirms its suitability in detecting both simple (origin hijacks) and man in the middle attacks (PJSC). Furthermore, it is able to flag hijacks regardless of their impact size, i.e. how many prefixes were affected.

F. Prefix hijack data

The central role that BGP plays have motivated researchers and practitioners to build systems for monitoring its stability. Notable in this respect are two projects, the University of Oregon's Routeviews project [2] and the RIPE NCC RIS project [3]. These two projects involve a large number of BGP speaking servers, called collectors, which maintain BGP connectivity to hundreds of routers in operational ASes. The collectors receive RIBs from these routers as well as all incremental updates. Recently, the BGPStream project at CAIDA has aggregated data from all collectors and made it available to the research community via a simplified interface. In this paper, we use RIBs snapshots and update traces made available by BGPStream. A RIB entry or a route announcement includes a field that shows the AS path followed to reach that entry. For example, in Fig S3 AS1 will reach a prefix p announced by AS 4 by following the path AS 3, AS 4, while AS 2 reaches a prefix p_1 announced by AS 7 potentially via the route AS 6, AS 7. The AS path field informs about the route to a prefix as well as the origin AS that is the last AS hop on the path. We collected the following datasets:

- *Malaysia Telekom incident.* We collected all the paths that went through Malaysia Telekom AS during the hijack (i.e. between 08:40 AM and 10:40 AM GMT on the 12th of June 2015), which amounted to 59031666 paths. For the data before the hijack, we collected the same paths during the same period on the previous day. These amounted to 757697 paths. To get these paths, we queried BGPStream for all available data for the respective time period and then extracted all paths that crossed ASN4788.
- *PJSC Rostelecom incident.* We collected all the paths that went through PJSC Rostelecom during the hijack on the 26th of April 2017, which amounted to 622137 paths. For the data before the hijack, we collected the same paths during a period of four hours from 2:40 PM to 6:40 PM on the 25th of April 2017, which amounted to 5530606 paths. We used four hours to get a stable view of all paths that were routed through PJSC before the hijack. Note that the hijack lasted only seven minutes.
- *MainOne incident.* We collected all paths that had both Google and MainOne ASNs (15169 and 37282) during the hijack, which amounted to 169 paths. We collected also all routes that crossed MainOne in the four hours between 6:00 PM and 10:00 PM on the 11th of November 2018. These amounted to 152518 paths.

Note that these paths included duplicates since some of the routers that BGPStream collects data from can belong to the same AS. Hence, we filtered the collected paths, which reduced the total number of paths significantly. Malaysia Telekom before and during the hijack paths were reduced to 20,914 and 432,783, respectively. PJSC before and during the hijack paths were reduced to 453,975 and 13,877, respectively. MainOne before and during hijack paths were reduced to 14,493 and 22, respectively.

G. Existing methods to detect prefix hijacks

Methods to address the vulnerabilities of the BGP routing can be naturally split into two categories. Methods of the first category are often referred to as *pro-active*, as they aim to substantially redesign BGP routing [15–19]. The efficiency of these methods requires their global adoption, which is not easy due to technical and financial costs [56]. Methods in the re-active category, on the other hand, aim to detect and mitigate BGP routing anomalies without altering the BGP protocol. These methods employ a variety of approaches to detect and mitigate BGP routing anomalies, ranging from active measurements to graph-theoretic and machine learning techniques [20–27]. Despite the variety of developed BGP routing anomaly defense methods, there are no methods that are simultaneously: (i) capable of detecting of all types of routing anomalies, (ii) scalable, and (iii) interpretable. We lack strategical planning tools that would allow ASes to estimate the risk their traffic will pass undesired ASes. Likewise, we are missing methods to forecast the changes in traffic patterns, given a specific perturbation to the AS Internet topology, e.g., a BGP hijack attack or a routing misconfiguration.

---

[2] http://www.routeviews.org/routeviews/
[3] https://www.ripe.net/analyse/internet-measurements/routing-information-service-ris

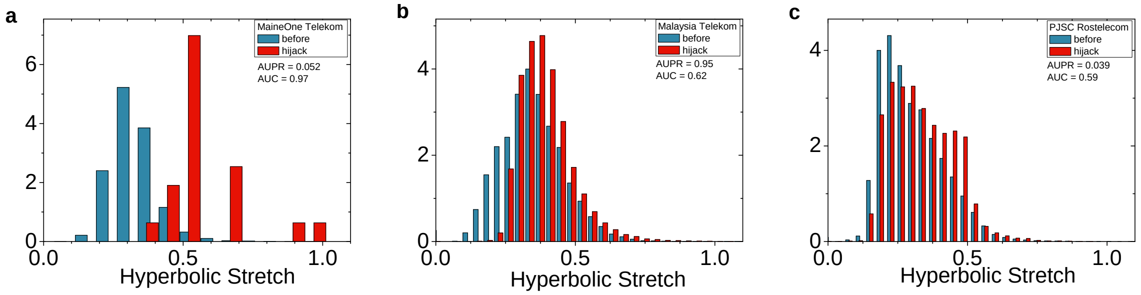

FIG. S4: Distributions of hyperbolic stretch values for BGP update paths observed before and during, **a**, MainOne Telekom, **b**, Malaysia Telekom and, **c**, PJSC Rostelecom prefix hijack events.

H. Hyperbolic stretch as a measure of path conformity

Since BGP routing paths are nearly shortest in terms of their network-based length, and shortest paths in the Internet tend to align along hyperbolic geodesics, we hypothesise that genuine routing paths should also align along corresponding geodesic. To assess the extent of such an alignment (or path conformity) we propose the following measure, which we refer to as the hyperbolic stretch. The hyperbolic stretch $D_{\Omega(A,B)}$ of path $\Omega(A,B)$ evaluates distance to geodesic $\gamma(A,B)$ of every node $C \in \Omega(A,B)$ and uses the maximum value:

$$D_{\Omega(A,B)} = \max_{C\in\Omega(A,B)} \frac{d(C,\gamma(A,B))}{d(A,B)} = \max_{C\in\Omega(A,B)} \frac{1}{2}\left[\frac{d(A,C)+d(B,C)}{d(A,B)} - 1\right], \tag{55}$$

where $d(A,B)$ is the length of geodesic $\gamma(A,B)$. The normalization by $d(A,B)$ allows one to compare stretches of paths with different endpoints. Note that in Eq. (55) we omitted the constant factor of $\ln 2$ in the expression for $d(C,\gamma(A,B))$. This is justified since the hyperbolic stretch is only used for ranking purposes.

We find the hyperbolic stretch values for BGP paths announced before the hijack event tend do be smaller than those for the BGP paths announced during the hijack events, Fig. 4 and S4. To quantify the possibility of inferring fake BGP paths, we mark BGP paths announced during the hijack event as true event and try identifying then by prioritizing BGP paths with largest hyperbolic stretch values. To quantify the classification accuracy we use the area under the Receiver-Operating Characteristic (AUC) and the area under the Precision-Recall curve (AUPR), obtaining AUPR = 0.052 and AUC = 0.97 for the MainOne hijack, AUPR = 0.95 and AUC = 0.62 for the Malaysia Telekom hijack, and AUPR = 0.039 and AUC = 0.59 for the PJSC Rostelecom hijack. Note that the AUPR value obtained for the Malaysia Telekom hijack is substantially higher than those obtained for the MainOne and the PJSC Rostelecom hijack. This is the case since the number of BGP paths announced during the Malaysia Telekom hijack is substantially larger than that announced before the event.

Justification for the hyperbolic stretch. At first glance, it seems that a more natural choice for the stretch would be either the latent geometric distance accumulated along the path or the average distance to geodesic from path nodes. The latent geometric distance accumulated along the path, upon closer consideration, appears to depend on the number of nodes in it. Indeed, since routing paths do not perfectly align along geodesics, each node in the path is located at certain non-zero distance to the geodesic. The sum of these distances correlates with the number of nodes in the path, while the length of the geodesic does not. As a result, longer routing BGP paths will be discouraged.

The average distance to geodesic is also suboptimal. In many circumstances, hijacked routing paths may contain only one non-genuine AS number. In such paths, therefore, only one node is substantially far from the geodesic. As a result, the average distance to geodesic, calculated using all nodes in the routing path, may turn out rather small.

Advantages of the hyperbolic stretch. Compared to existing prefix hijack detection methods, discussed in Section V G, the hyperbolic stretch has several promising advantages. In order to calculate the hyperbolic stretch of a path, one needs to know all latent-geometric coordinates of the AS nodes. Once these coordinates are known, the calculation of the hyperbolic stretch is a straightforward computational task. Latent geometric coordinates of ASes are found through the process of Internet mapping, as discussed in Section IV. Even though the process Internet mapping does require the global knowledge of the Internet topology, it is not sensitive to missing information and the intermittent dynamics, as demonstrated in the main text. A previous work on hyperbolic mapping of the Internet estimates that given the current growth rate of the Internet, one needs to produce the new map once a decade, while coordinates of new ASes can be computed locally [34]. Thus, latent geometric coordinates of ASes can be computed and distributed by a central authority. Since AS coordinates are semi constant, we do not foresee the need to communicate them along with BGP paths. The calculation of the hyperbolic stretch does not carry significant

computational overhead, in contrast to active monitoring methods. Compared to machine-learning anomaly detection methods, the hyperbolic stretch is interpretable, allowing ASes to explain their routing decisions.

Limitations and further work. While the hyperbolic stretch is a promising measure of BGP path conformity, we emphasize that further research is needed to properly assess its utility in the detection of BGP prefix hijacks.

On the one hand, BGP routing in general is affected by the policies and business relationships established by ASes [48]. As a result, plausible BGP paths that comply with AS policies and business relationships are not necessarily shortest and, therefore, may not fully align along hyperbolic geodesics. On the other hand, empirical studies, establish that real BGP paths are, nevertheless, short and close to optimal [57]. Thus, a more systematic analysis of the hyperbolic stretch of genuine BGP paths is needed.

Ultimately, the hyperbolic stretch needs to be converted into the decision of path inclusion into the RIB table. The most straightforward approach, to this end, is to institute a cut-off value for the maximum admissible hyperbolic stretch. Thus, further research into the choice of the optimal cut-off value is needed.

On a fundamental level, the hyperbolic stretch should only be regarded as the first step towards using the hyperbolic geometry in BGP prefix hijack detection. The hyperbolic stretch is computed as the maximum distance to the geodesic from the path nodes. Instead of using the maximum distance to geodesic value, one could benefit from using the entire set of the distances, e.g, as a distribution of distance to geodesic values. This distribution can be compared, using statistical tests, to those of genuine paths received earlier.

VI. PROTEIN-PROTEIN INTERACTION NETWORK AND CELLULAR PATHWAYS

A. Protein interaction data

As the first step to construct a unified human interactome, we resort to two different protein-protein interaction (PPI) data sources: High-quality INTeractomes database (HINT) [58], and Human Reference Protein Interactome Mapping Project (HuRI) [30]. HINT (http://hint.yulab.org) is organized as a centralized database of high-quality human PPIs integrated from several other databases and annotated using both, an automated protocol and manual curation. Unlike the HINT database, HuRI is a primary source for experimentally validated PPIs using yeast-two-hybrid experiments. The two PPI sources were considered because they provide complementary views of the whole human interactome. The current release of HINT database contains $45,517$ interactions excluding self-loops. For the HuRI dataset, we collect $31,653$ unique interactions excluding self-loops. In total, we generate a human interactome consisting of $63,175$ interactions, where $13,995$ interactions exist in both data sources. The largest connected component of the combined protein-protein interaction network includes $11,970$ proteins that are involved in $63,118$ physical interactions.

B. Similarity-based representation of protein interactions

Since latent-geometric distances are interpreted as measures of node similarities, our second step is to extract the similarity-based representation of the PPI network. This step is necessary since interacting proteins are not necessarily similar. Instead, interacting proteins often have complementary properties [59–64]. Therefore, to identify pairs of similar proteins, we create a dual PPI network, where protein pairs are connected if they have common interaction partners in the original network, with link weights equal to the number of common interaction partners in the original PPI network, Fig. S5**a,b**. We refer to the resulting network as similarity-based. The resulting similarity-based dual network is extremely dense, consisting of $11,970$ proteins and $2,674,367$ weighted connections, corresponding to the average degree of $\langle k \rangle = 446.8$. The largest link weight is $\omega_{\max} = 253$.

Therefore, to obtain meaningful embedding of the resulting network we extract its sparse backbone consisting of only significantly large weights.

One approach to filter insignificant weights could be to impose a global threshold on link weights in the network and keep only links with weights exceeding this threshold. At the second glance, however, this procedure would turn suboptimal since large link weights are correlated with the node degrees, and removing links with small weights would lead to pruning the network of small-degree nodes.

Instead of using the global cutoff, we utilize the alternative filtering procedure, known as the *disparity filter* [65]. The disparity filter tailors the weight cutoff value according to the local neighborhood of a given link, and has been shown to work well in weighted networks with heterogeneous distribution of weights. For the reader's convenience we outline the basic computational steps of the disparity filter here and invite to study the original work, Ref. [65], for further details.

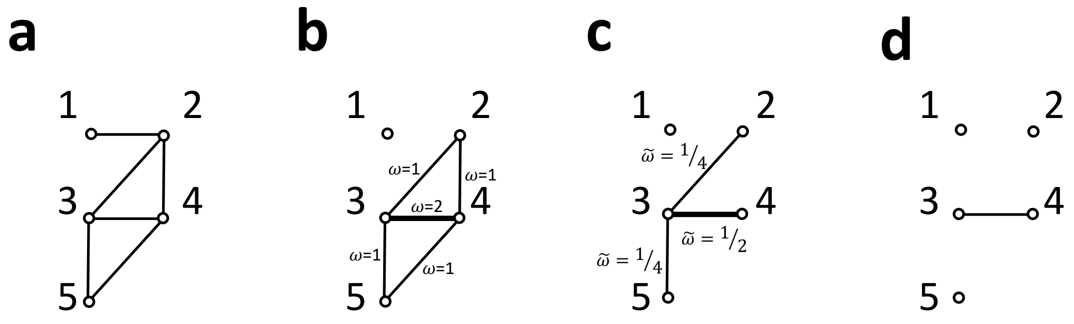

FIG. S5: **Schematic extraction of the similarity backbone of the toy protein interaction network**. Panel **a** depicts the original toy network of protein interactions. Panel **b** corresponds to its dual similarity-based representation where link weights correspond to the number of interaction partners between corresponding proteins. Shown in **c** are the normalized link weights adjacent to protein 3. The probability that links weights are generated by chance, given by Eq. (59), are $\alpha_{32} = \alpha_{35} = 0.5625$ and $\alpha_{35} = 0.25$. Panel **d** depicts the resulting backbone of the similarity-based network, obtained with the cutoff $\alpha = 0.5$.

Disparity filter, Ref. [65] To identify links with weights significantly larger than expected by chance, for every node i in the dual similarity-based networks we examine all links $\{ij\}$ adjacent to it. For all $\{ij\}$ links adjacent to node i we first compute normalized weights as

$$\tilde{\omega}_{ij} = \frac{\omega_{ij}}{\sum_{j \in \Omega_i} \omega_{ij}}, \tag{56}$$

where Ω_i is the set of neighbors of node i. We denote the degree of node i as $k_i = |\Omega_i|$, Fig. S5**c**. To determine the significance of the normalized link weight $\tilde{\omega}_{ij}$, we evaluate the probability that $\tilde{\omega}_{ij}$ is generated by chance by distributing unit weight among k_i neighbors of node i. This problem is equivalent to the distribution of $k_i - 1$ points on a unit interval. The probability density function for the gap between two adjacent points is

$$\rho_\omega(x) = (k_i - 1)\,(1 - x)^{k_i - 2}\,, \tag{57}$$

and the probability that a randomly chosen gap has size larger than x_0 is

$$\int_{x_0}^{1} \rho_\omega(x)\mathrm{d}x = (1 - x_0)^{k_i - 1}\,. \tag{58}$$

Similarly, the probability for a normalized link weight larger than $\tilde{\omega}_{ij}$ to be generated by chance among k_i links is

$$\alpha_{ij} = (1 - \tilde{\omega}_{ij})^{k_i - 1}\,. \tag{59}$$

Then, link weight $\tilde{\omega}_{ij}$ is considered significant if α_{ij} is less than certain cutoff value,

$$\alpha_{ij} < \alpha, \tag{60}$$

see Fig. S5**c**. Having identified links with statistically significant weights one extracts the unweighted backbone of the network by keeping only links with statistically significant weights: any link ij is preserved if its weight ω_{ij} is statistically significant with respect to either node i and j, Fig. S5**d**. The selection of α is network-specific. In general, selecting very smaller α results in the removal of a larger fraction of links, which leads to a sparser network with a smaller size of the largest connected component. On the other had, the selection of larger cutoff value α results in a denser network with structural properties similar to the original weighted network.

In our analysis we set $\alpha = 0.1$. For brevity we refer to the resulting unweighted network as the similarity-based PPI network. The largest connected component of the similarity-based PPI consists of $N = 5,191$ proteins connected with $E = 156,191$ similarity links, spanning 43% of the original PPI network. We emphasize that connection in the resulting network reflect similarities between proteins and do not correspond the physical interactions.

C. Mapping the similarity-based protein interaction network

To embed the resulting similarity-based PPI network to the hyperbolic disk we utilize the HyperLink Embedder [45], that we developed in our earlier work. Being based on the MLE optimization, the HL Embedder tends to map large

degree nodes close to each other in the $\mathbb{H}^2$. To avoid this effect we disregard connections in the network between nodes with degrees larger than 500. Finally, since the embedding is only possible for the connected component of the graph, we extract the largest connected component of the unweighted similarity-based PPI network, obtaining $N = 4,972$ proteins and $E = 107,303$ similarity relations.

The similarity-based PPI backbone network is characterized by a fat-tail degree distribution, which we assume to be scale-free for embedding purposes, $P(k) \sim k^{-\lambda}$ with the exponent of $\lambda = 2.1$. Average degree-dependent clustering coefficient of the network is $\bar{c} = 0.52$, corresponding to the embedding temperature of $T = 0.4$.

D. Latent-geometric alignment and genetic composition of cellular pathways

We consider three cellular pathways: transforming growth factor beta (TGFb), cell cycle pathway, and ubiquitin proteasome pathway (UPP), which we extracted from the Kyoto Encyclopedia of Genes and Genomes (KEGG) [66]. After the data extraction, we mapped the obtained pathway gene sets to the similarity-based PPI network. Of 83 genes comprising the TGFb pathway, 56 are present in the GCC of the similarity-based PPI network. In the case of the UPP pathway, of 137 genes 95 are present in the GCC of the similarity-based PPI network. Finally, of 124 genes in the cell cycle pathway, 103 are present in the GCC of the similarity-based PPI network.

Figure S6 depicts pathway proteins on the hyperbolic map of the similarity-based PPI network. We found that these cellular pathways are localized in the hyperbolic space, and their localization patterns resemble hyperbolic geodesics, see Fig. S6. We next looked into the genetic composition of each pathway. Each of these pathways had subsets of genes clustered along two branches. Surprisingly, all three pathways are localized within the fourth quadrant of the hyperbolic disc, Fig. S6. The functional analysis of each of the six clustered subsets revealed that the proteins in five out of six subsets shared certain functional similarity with each other.

In the UPP, a subset corresponding to the geodesic branch at 0 degree is exclusively associated with E3 ubiquitin-protein ligase, one of the three classes of enzymes involved in protein ubiquitination and degradation. For the larger subset clustered around geodesic at 300 degrees, we found that the gene were associated with E2 and E3 classes of enzymes, but not with E1, Fig. S6**a**. E1s are ubiquitin-activating enzymes and E2s are ubiquitin-conjugating enzymes [67].

In case of the TGFb pathway, genes clustered around the geodesic line at 0 degrees were all associated with either transcription factor or tyrosine kinase activities, while genes clustered around geodesic line at 300 degrees are exclusively associated with SMAD regulatory activity and includes receptor-regulated SMADs and their receptors as well as inhibitory SMADs [68], see Fig. S6**b**.

Finally, in the most general, cell cycle, pathway a relatively small subset clustered around geodesic at 300 degrees corresponded exclusively to seven known isoforms of YWHA as well as CDC25B which YWHA binds to and subsequently sequesters [69]. The subset clustered around 0 degrees was not associated with one specific function and had groups of proteins linked to different stages of cell cycle.

E. Genes in the latent-geometric vicinity of the UPP pathway

To understand the role of genes in the neighborhood of the pathway we considered 100 genes in the latent-geometric proximity to the UPP pathway, as quantified by the distance to the fitted geodesic, Eq. (31). We used DAVID tools [70], a bioinformatics framework specifically designed to provide systematic functional analysis for large group of genes, to functionally cluster groups of the proximal genes independently from the genes in the geodesic cluster, finding 6 major clusters, Fig. S7. As expected, the most highly ranked cluster contained the terms related to ubiquitination, cluster 1 in Supplementary Data 3, Fig. S7**a**. Interestingly, the other clusters include gene associated with the immune response signaling pathway, cluster 2 (Fig. S7**b**), viral infections, clusters 3 (Fig. S7**c**) and 5 (Fig. S7**d**), pathways associated with several types of cancer, clusters 3 and 4, as well as zinc fingers, cluster 5 (Fig. S7**e**), and DNA repair, cluster 6 (Fig. S7**f**) in Supplementary Data 3. Each of the functional groups is naturally associated with ubiquitination [71–75]. Indeed, ubiquitination has been well-known as a key mechanism regulating signal transduction and mediating both innate and adaptive immune responses [71]. On the other hand, the principal role of protein containing zinc finger domains in ubiquitination has emerged only recently [76–79]. These findings thus illustrate a potentially new way of discovering functional links between the well-characterized pathways and groups of genes that are not directly involved in them.

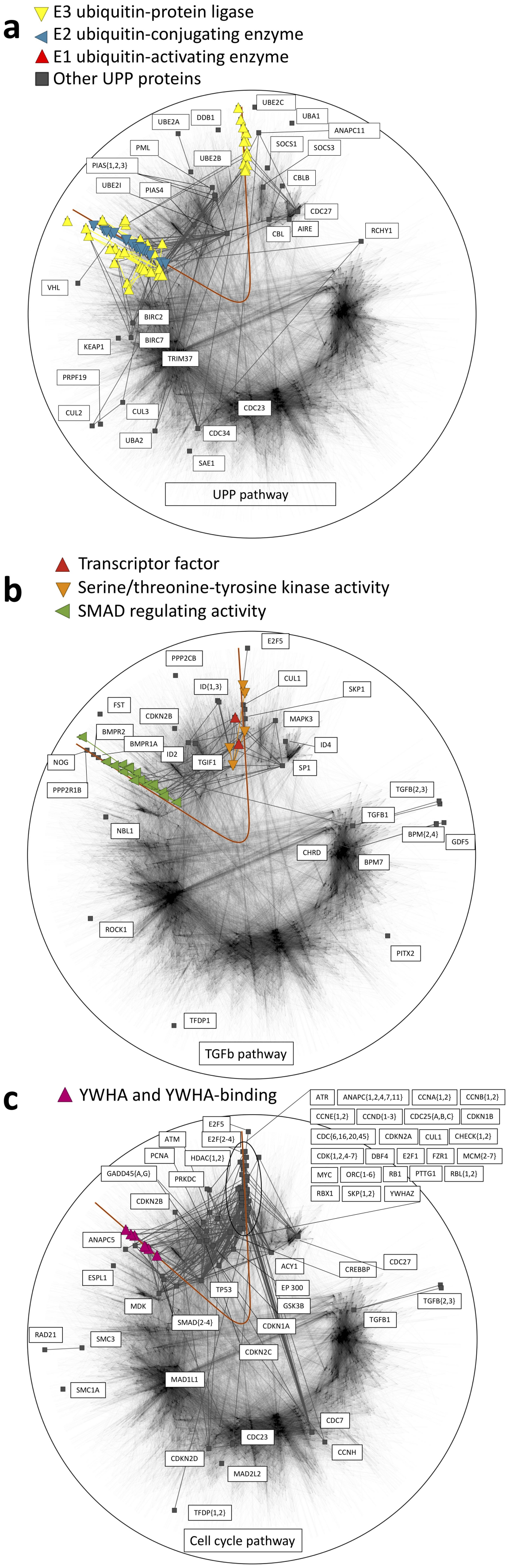

FIG. S6: Cellular pathways on the hyperbolic map of the similarity-based PPI network. Shown are, **a**, UPP, **b**, TGFb, and, **c**, cell cycle pathways. Points correspond to pathway proteins, interactions are not shown for better readability. Solid lines are fitted hyperbolic geodesics.

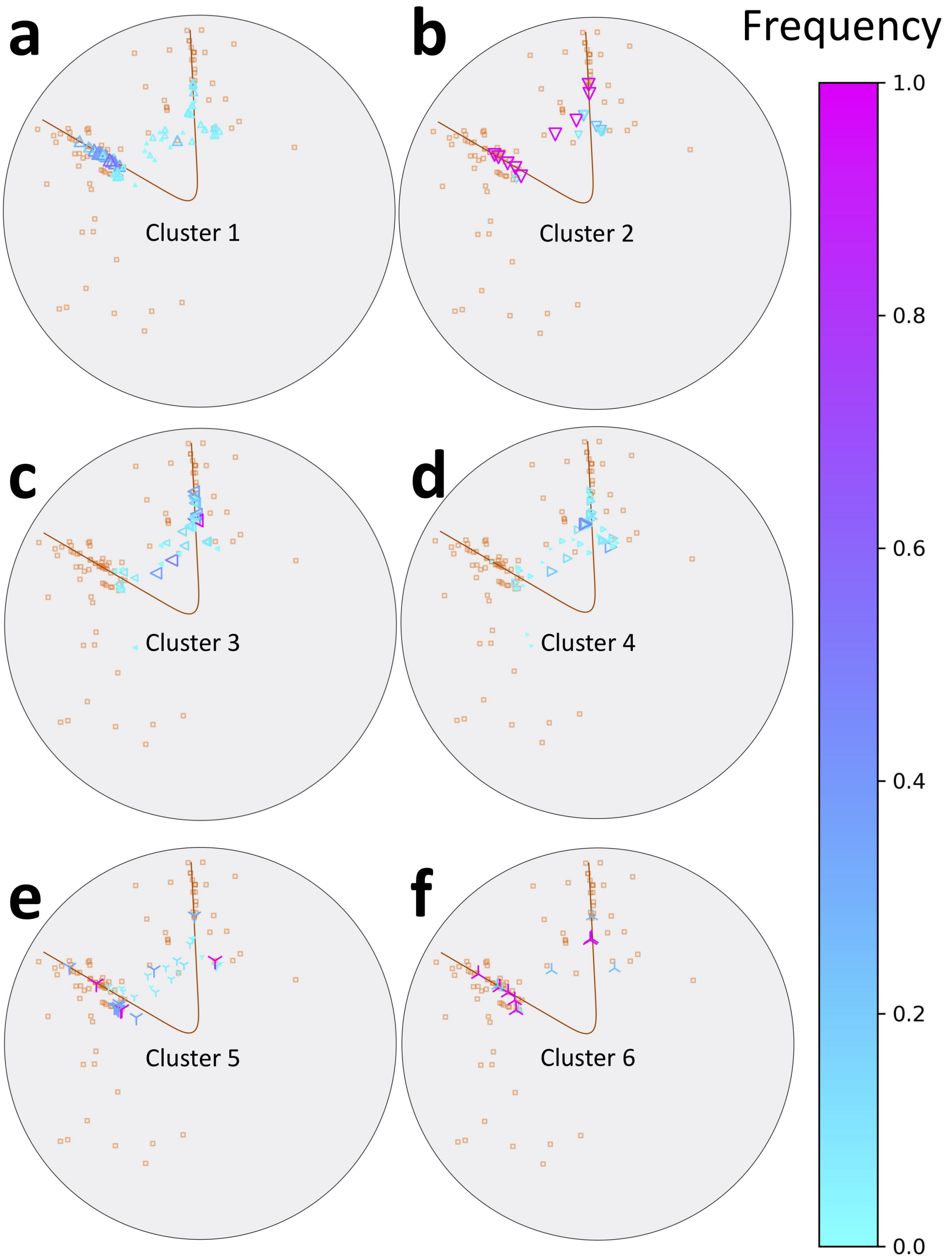

FIG. S7: 6 gene clusters in the geometric vicinity of the UPP geodesic. UPP pathway proteins are shown with orange squares. The solid line is the fitted UPP hyperbolic geodesic. Since each cluster is represented by several genes sets corresponding to different annotation sources, we color-code genes based on the fraction of gene sets they belong to.

VII. PRETTY-GOOD-PRIVACY (PGP) WEB OF TRUST NETWORK

PGP is a data encryption and decryption computer program that provides cryptographic privacy and authentication for data communication [80]. The data are collected and maintained by Jörgen Cederlöf [81]. In the paper, we use the PGP snapshot taken in April 2003. The PGP web of trust is a directed network where nodes are certificates consisting of public PGP keys and owner information. A directed link in the web of trust pointing from certificate A to certificate B represents a digital signature by the owner of A endorsing the owner/public key association of B. We construct the undirected PGP graph by taking into account only bidirectional trust links between the certificates. Further, we only consider the giant connected component of the resulting undirected PGP web of trust network.

As a result of keeping only bidirectional links, we increase the chance that the owners of PGP certificates know each other, making the resulting network a proxy for the social trust network between PGP users.

The resulting PGP web of trust network has $N = 14,138$ certificates (nodes) and $E = 160,080$ connections. PGP web of trust is characterized by a scale-free degree distribution with exponent $\gamma = 2.1$ and the average degree-based clustering coefficient of $\bar{c} = 0.60$, corresponding to RHGs with temperature $T = 0.8$.

VIII. IDENTIFICATION OF NEARLY SHORTEST PATH NODES: DEFINITIONS AND METRICS

Nearly shortest path nodes. We define nearly shortest path nodes as nodes that become shortest path nodes with probability exceeding 0.05 if network links are removed uniformly at random with rate $q = 0.5$. Our definition of nearly shortest path nodes is motivated by the observation that communication paths do not have to be strictly shortest. Further, due to the dynamic nature of communication networks shortest paths connecting the node pair of interest are subject to change, and a node that does not belong to a shortest path at time t_1, may be come a shortest path node at time $t_2 > t_1$.

Given the origin-destination A-B node pair, we compute nearly shortest path nodes as follows. We conduct $1,000$ independent simulations. In each simulation, $\ell = 1, ..., 1000$, we remove network links uniformly at random with the rate of $q = 0.5$, obtaining incomplete network $\tilde{G}_\ell$. We next make sure that both origin and destination nodes A and B belong to the giant connected component (GCC) of $\tilde{G}_\ell$. If this is not the case, $\tilde{G}_\ell$ is discarded and another instance of $\tilde{G}_\ell$ is generated. We next identify the set of shortest path nodes connecting A with B in $\tilde{G}_\ell$ and denoted this set as $\Omega_\ell(A, B)$. After completing the simulation we compute for each node of the network the fraction of time the node was observed in the shortest path connecting A-B nodes:

$$p_i(A, B) = \frac{\sum_{\ell=1}^{1000} \mathbb{1}_{\Omega_\ell(A,B)}(i)}{1000}, \tag{61}$$

where $\mathbb{1}_\Omega(i)$ is the indicator function, which equal unity if i belongs to set Ω, and is zero otherwise. We interpret $p_i(A, B)$ as the probability that node i belongs to the shortest path $\Omega_\ell(A, B)$ under random perturbation of network topology. Nearly shortest path nodes are defined as those with $p_i(A, B) > 0.05$.

Precision score. In our work we utilize the distance to geodesic to identify nearly shortest path nodes: the smaller the distance to geodesic the higher is the chance that the node of interest belong to the set of nearly shortest path nodes. To quantify the accuracy of the distance to geodesic, we use the statistical precision score, which we compute as follows. Given the A-B node pair, we first compute distance from every other network node C to geodesic $\gamma(A, B)$ using Eq. (31). Second, we compile the list of candidate nodes Ω_M, sorted in the increasing order of their distance to $\gamma(A, B)$ geodesic ranking. We denote the fraction of $\lambda \in [0, 1]$ of these nodes as set $\Omega_M(\lambda)$. Third, we compute the ground-truth set Ω_R of nearly-shortest path nodes using the original unperturbed network G.

The precision score quantifies the intersection between $\Omega_M(\lambda)$ and the ground-truth Ω_R:

$$\text{Precision} = \frac{|\Omega_R \bigcap \Omega_M(\lambda^*)|}{|\Omega_R|}, \tag{62}$$

where λ^* corresponds to $|\Omega_M(\lambda^*)| = |\Omega_R|$. We compare the precision of distance to geodesic to that of the network-based method. To compute the precision of the network-based method, we replace distance to geodesic values with those of network based distances.

Number of steps to disrupt all paths. This metric quantifies the importance of the identified A-B pathway nodes by calculating the required number of node removals, in the decreasing order of their relevance, necessary to fully disrupt the communication between A and B: the smaller the number of node removals the more crucial are the identified nodes. To compute this score we remove nodes one-by-one in the increasing order of distance to geodesic $\gamma(A, B)$ until there is exists no path connecting nodes A and B.

A. Network-based and random-walk based path finding metrics.

In our work, we compare the accuracy of distance to geodesic to several alternative metrics.

To benchmark the performance of the distance to geodesic metric we employ several network-based methods.

Network-based metric: $d_{\rm nb}(C|A,B) = \ell_{A,C} + \ell_{C,B}$, where $\ell_{X,Y}$ is the shortest path distance between nodes X and Y. $d_{\rm nb}(C|A,B)$ is minimized when C lies on a shortest path between A and B. The larger $d_{\rm nb}(C|A,B)$ the less the relevance of C to paths between A and B.

Random-walk-based metric 1*:* $d_{\rm comm}(C|A,B) = n(A,C) + n(C,B)$, where $n(X,Y)$ is the average commute time between X and Y. $n(X,Y) \equiv m(X|Y) + m(Y|X)$, where $m(X|Y)$ is the mean first passage time from Y to X [82] .

To compute the average commute times efficiently we rely on the result of Ref. [83] relating $n(X,Y)$ to the pseudoinverse of the network Laplacian matrix. The Laplacian matrix L is defined as

$$L = D - A, \tag{63}$$

where A is the network adjacency matrix and D is the degree diagonal matrix, $D_{ij} \equiv k_i \delta_{ij}$. Here k_i is the degree of node i and δ_{ij} is the Kronecker delta function. The Moore-Penrose pseudoinverse [84] of laplacian L^+ is unique and satisfies

$$LL^+L = L. \tag{64}$$

Given L^+, average commute times can be computed as

$$n(i,j) = V_G \left(L^+_{ii} + L^+_{jj} - 2L^+_{ij} \right), \tag{65}$$

where $V_G = \sum_{\ell=1}^{N} k_\ell$.

In our work, we were able to compute the pseudoinverse of the Laplacians for the similarity-based PPI and the PGP web of trust networks but not for the Internet due to its large size. Hence, we included $d_{\rm comm}(C|A,B)$ scores in the analyses of pathfinding accuracies of the similarity-based PPI and the PGP web of trust networks.

Random walk-based metric 2*:* $d_{\rm rw}(C|A,B)$ is the random walk hit frequency. To compute this metric, we initiate M independent random walks from nodes A and B of fixed length D, counting the number of times random walks visits C from A and B. In our simulations we used $M = 1,000$ and $D = 20$ in our simulations.

B. Path finding on synthetic networks.

We conduct path finding experiments on RHG networks with two goals in mind. On the one hand, we treat these tests as benchmarks for our path reconstruction strategies. On the other hand, these tests build our intuition and help us interpret path reconstruction results on real networks.

In the following we generate RHGs of $N = 5,000$ nodes each, $\langle k \rangle = 10$ and variable $\lambda \in [2.1, 2.9]$ and $T \in [0.1, 0.9]$ parameters, which we increment with steps of $\Delta\lambda = 0.1$ and $\Delta T = 0.1$, respectively.

For each set of λ-T parameters we build one RHG realization $G(\lambda,T)$. We **forget** the original node coordinates of $G(\lambda,T)$. Given $G(\lambda,T)$, we then obtain its incomplete instance $\tilde{G}(\lambda,T)$ by removing each of its links independently with rate $q = 0.5$. Next, we proceed to map the largest connected component of $\tilde{G}(\lambda,T)$ to the 2-dimensional hyperbolic disk $\mathbb{H}^2$ using the HL embedder.

After obtaining the hyperbolic map of $\tilde{G}(\lambda,T)$ we compile lists of node pairs for path finding experiments. We obtain two node pair cohorts, such that node pairs in each cohort are separated, respectively, by angles $\Delta\theta = \frac{\pi}{2}$ and $\Delta\theta = \frac{\pi}{8}$. For each, $\Delta\theta$ value we consider all possible node pairs of degree $k = 5$ each in G each. For each ij node pairs we calculate its angular separation $\Delta\theta_{ij} = \pi - |\pi - |\theta_i - \theta_j||$ in $\mathbb{H}^2$. We then rank all node pairs in the increasing order of $|\Delta\theta_{ij} - \Delta\theta|$. Next, we exclude from the list all node pairs that are either not part of the largest connected component of $\tilde{G}(\lambda,T)$ or directly connected to each other in $\tilde{G}(\lambda,T)$. We define cohort $C_{\Delta\theta}$ for our path finding experiments to consist of 100 node pairs from the list with the smallest $|\Delta\theta_{ij} - \Delta\theta|$ values.

After obtaining both the map of $\tilde{G}(\lambda,T)$ and node pair cohort $C_{\Delta\theta}$, we proceed with path finding experiments. To this end, we consider every i-j node pair in $C_{\Delta\theta}$. For each node pair, we compute the ground-truth nearly shortest path nodes in $G(\lambda,T)$, we obtain shortest path node candidates based on (i) distance to geodesic in $\tilde{G}(\lambda,T)$ and (ii) network-based distance in $\tilde{G}(\lambda,T)$. We use these rankings to calculate the statistical precision scores for each i-j pair in $C_{\Delta\theta}$. For $C_{\Delta\theta}$ we report its average precision score and its standard deviation. To compute the number of node removal steps we remove nodes one in the order set by either distance to geodesic or network-based distance until there is no paths connecting node pair i-j in $G(\lambda,T)$. For $C_{\Delta\theta}$ we report its average number of node removals and its standard deviation.

The results of our path finding tests are presented in Figs. S8, S9, S10, and S11. All results indicate that the accuracy of path finding using the distance to geodesic is nearly independent of the scale free degree distribution exponent $\lambda \in (2,3)$. At the same time, the accuracy of the distance to geodesic is dependent on HRG geometricity, which is controlled by temperature T. The smaller T is the higher is the geometricity and, as a result, the higher is the accuracy of distance to geodesic. The accuracy of the network-based method in finding nearly shortest path nodes is consistently lower than that of the distance to geodesic, and is nearly independent of both λ and T.

C. Path finding on incomplete networks: the similarity-based PPI network and the PGP web of trust

Path finding experiments on the similarity-based PPI network and the PGP web of trust are designed similar to those on synthetic networks.

We start with the original network G. For the given missing link rate $q = \{0, 0.3, 0.5, 0.7, 0.9\}$ we next obtain 1 instance of the incomplete network $\tilde{G}$. To do so, we remove every network link in G independently with probability q, and then extract the largest connected component from the remaining network, obtaining $\tilde{G}(q)$. We next apply the HL embedder to map $\tilde{G}(q)$ to the 2-dimensional hyperbolic disk $\mathbb{H}^2$.

After obtaining the hyperbolic map of $\tilde{G}(q)$, we compile a random cohort C of $1,000$ node pairs for path finding experiments. To do so, we draw $1,000$ node pairs at random with replacement from the largest connected component of $\tilde{G}(q)$. We disregard node pairs that are directly connected in $\tilde{G}(q)$.

After obtaining both the map of $\tilde{G}(q)$ and cohort C, we proceed with path finding experiments. To this end, we consider every i-j node pair in C. For each node pair, we compute the ground-truth nearly shortest path nodes in the original network G. We obtain shortest path node candidates based on (i) distance to geodesic in $\tilde{G}(q)$, (ii) network-based distance $d_{\rm nb}$, and random walk based metrics $d_{\rm comm}$ and $d_{\rm rw}$. We use these rankings to calculate the statistical precision scores for each i-j pair in C. We report the average precision scores and the average number of node removal steps needed to disconnect a pair of interest. To compute the number of node removal steps, we remove nodes in the order of their ranking until there are no paths connecting node pair i-j in G. We report path finding results for the similarity-based PPI network and the PGP web of trust in Figs. S12 and S12, respectively.

Since missing links (false negatives) often co-occur with spurious links (false positives) we next asked if our results hold when a small fraction of spurious links are present. To answer this question, we repeated our path finding experiments by first adding 10% of the missing links to the three networks of Interest. Our results in this case fully agree with those obtained for networks without spurious links, Fig. S14.

D. Path finding on networks with spurious links.

In this section, we test the accuracy of the distance to geodesic metric in finding nearly shortest path nodes when spurious links are present. Our experiments are designed similarly to those with missing links.

We start with the original network G. For the given spurious link fraction $q = \{0.3, 0.5, 0.7, 0.9\}$ we add qE spurious links to the network uniformly at random, obtaining a spurious network $\tilde{G}(q)$. Here, E is the number of links in G. We next apply the HL embedder to map $\tilde{G}(q)$ to the 2-dimensional hyperbolic disk $\mathbb{H}^2$.

The remaining steps are identical to those in the missing links experiments.

After obtaining the hyperbolic map of $\tilde{G}(q)$, we compile a random cohort C of $1,000$ node pairs for path finding experiments. To do so, we draw $1,000$ node pairs at random with replacement from the largest connected component of $\tilde{G}(q)$. We disregard node pairs that are directly connected in $\tilde{G}(q)$.

After obtaining both the map of $\tilde{G}(q)$ and cohort C, we proceed with path finding experiments. To this end, we consider every i-j node pair in C. For each node pair, we compute the ground-truth nearly shortest path nodes in the original network G. We obtain shortest path node candidates based on (i) distance to geodesic in $\tilde{G}(q)$, (ii) network-based distance $d_{\rm nb}$, and random walk based metrics $d_{\rm comm}$ and $d_{\rm rw}$. We use these rankings to calculate the statistical precision scores for each i-j pair in C. We report the average precision scores and the average number of node removal steps needed to disconnect a pair of interest. To compute the number of node removal steps, we remove nodes in the order of their ranking until there are no paths connecting node pair i-j in G.

As seen from Fig. S15, the accuracy of distance to geodesic remains sufficiently high in situations where spurious links are present. Different from the missing case, however, the accuracy of distance to geodesic is lower than that of the network-based method.

This is the case since the accuracy of the network-based method is not strongly affected by spurious links. Most nodes in considered networks – AS Internet, similarity PPI, and the PGP web of trust – are small-degree nodes, as

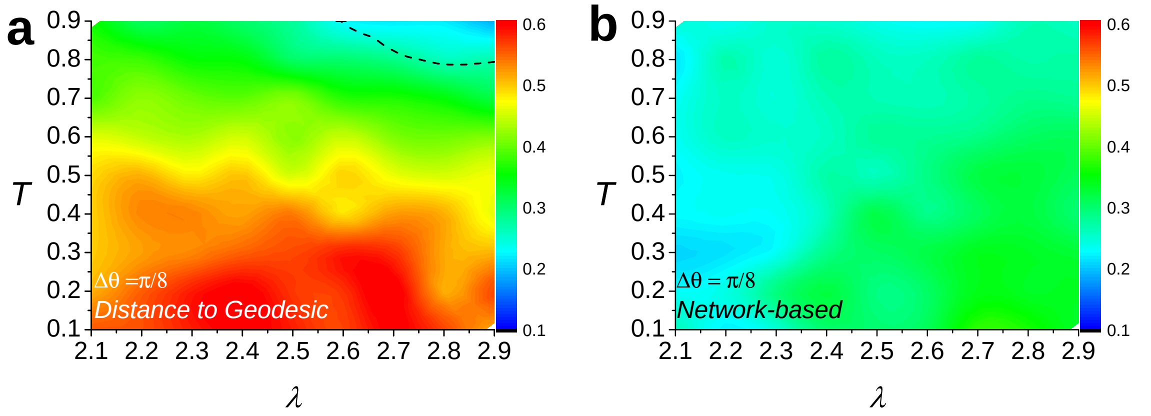

FIG. S8: **Identification of nearly shortest paths in incomplete networks.** We consider **a**, distance to geodesic and **b**, network-based strategy in incomplete networks. Heatmaps consist of $9 \times 9 = 81$ points, each corresponding to an RHG with $N = 5,000$, $\langle k \rangle = 10$, and different λ and T parameters. Color reflects the average precision for identification nearly shortest path nodes corresponding to the A_ℓ-B_ℓ pair. Each point is the average over all $\{A_\ell$-$B_\ell\}$ pairs, $\ell = 1, .., 100$ in the $\Delta\theta = \frac{\pi}{8}$ cohort. Note that the distance to geodesic strategy, panel **a** offers superior performance compared to the network-based strategy across nearly entire λ-T phase space. The distance to geodesic strategy performs best at lowest T values, which correspond to the highest network geometricity.

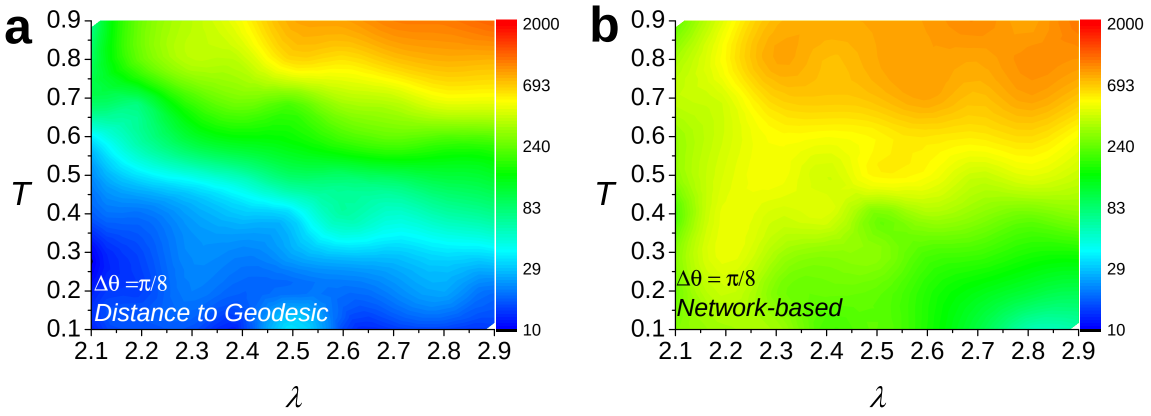

FIG. S9: **Disrupting paths in incomplete networks.** We consider **a**, distance to geodesic and **b**, network-based strategy in incomplete networks. Heatmaps consist of $9 \times 9 = 81$ points, each corresponding to an RHG with $N = 5,000$, $\langle k \rangle = 10$, and different λ and T parameters. Color reflects the average number of node removals necessary to disconnect a pair of nodes A_ℓ-B_ℓ of interest. Each point in the heatmap is the average over all $\{A_\ell$-$B_\ell\}$ pairs, $\ell = 1, .., 100$ in the $\Delta\theta = \frac{\pi}{8}$ cohort. Note that the geodesic proximity strategy, panel **a** offers superior performance compared to other considered strategies across nearly the entire λ-T phase space. The distance to geodesic strategy performs best at lowest T values, which correspond to the highest network geometricity.

reflected by their scale-free degree distributions. Therefore, paths selected uniformly at random tend to have small-degree endpoint nodes. By the same argument, spurious links added at random tend to connect small-degree nodes. As a result, randomly chosen paths are rarely affected by spurious links. Indeed, a path between two small-degree endpoint nodes tends to pass large degree nodes, which are statistically less likely to have randomly added spurious links. A path is only affected by a spurious link if it connects the immediate neighborhoods of its both endpoints.

In other words, randomly missing links are statistically different from randomly added spurious links. The former are selected at random from the set of existing links, while the latter are selected at random from the set of all unconnected node pairs. This observation makes us ask, are true spurious links fully random or governed by some other mechanisms, e.g., preferential attachment? This question is a part of a more general and challenging question: how can one identify paths when non-uniformly missing links are present? We hope to advance in solving this problem in the future.

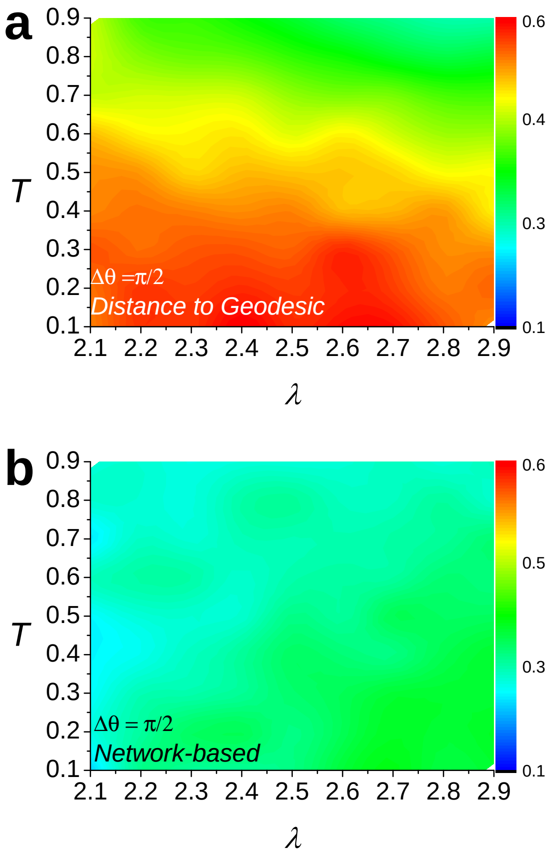

FIG. S10: Heatmaps quantify the average precision for the identification of communication paths in incomplete RHGs for the $\Delta\theta = \frac{\pi}{2}$ cohort. Parameters are identical to those in Fig. S8.

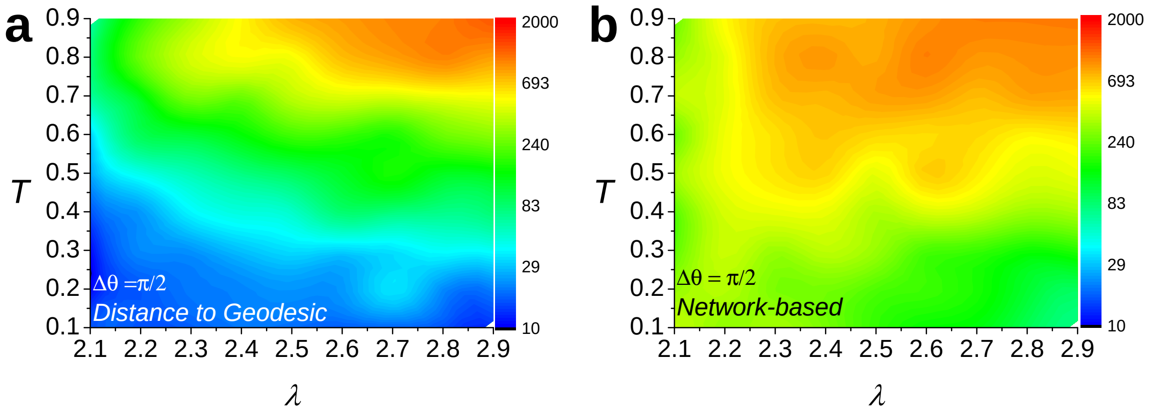

FIG. S11: Heatmaps quantify the average number of node removals necessary to disconnect pairs of nodes in incomplete RHGs with angular separation of $\Delta\theta = \frac{\pi}{2}$. Parameters are identical to those in Fig. S9.

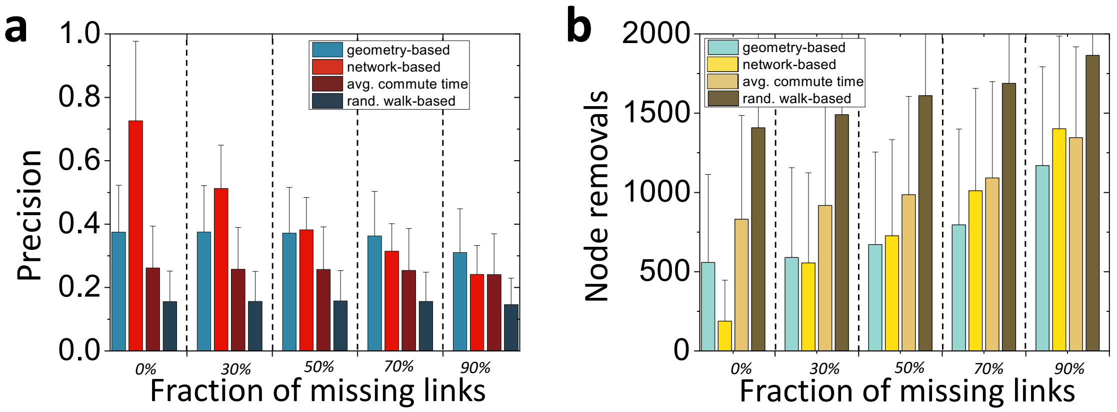

FIG. S12: The accuracy of path finding in the incomplete similarity-based PPI network. The accuracy is quantified by (**a**) statistical precision and (**b**) the number of node removals to disconnect a node pair of interest. Note that the accuracy of the network-based method decreases fast as the fraction of missing links increases. The accuracy of the distance to geodesic decreases at a slower rate that of the network-based method, exceeding it for $q > 0.5$.

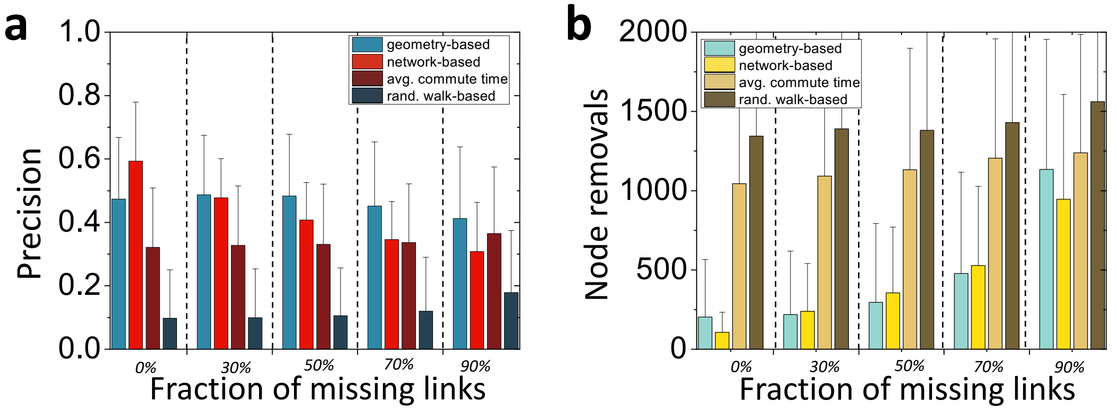

FIG. S13: The accuracy of path finding in the incomplete PGP web of trust network. The accuracy is quantified by (**a**) statistical precision and (**b**) the number of node removals to disconnect a node pair of interest. Note that the accuracy of the network-based method decreases fast as the fraction of missing links increases. The accuracy of the distance to geodesic decreases at a slower rate that of the network-based method, exceeding it for $q > 0.3$.

Supplementary References

[1] B. V. Cherkassky, A. V. Goldberg, and T. Radzik, *Shortest Paths Algorithms: Theory and Experimental Evaluation,* Math. Program. **73**, 129 (1996).

[2] E. W. Dijkstra, *A Note on Two Problems in Connexion with Graphs,* Numer. Math. **1**, 269 (1959).

[3] P. Wang, B. Xu, Y. Wu, and X. Zhou, *Link Prediction in Social Networks: the State-of-the-Art,* Sci. China Inf. Sci. **58**, 1 (2015).

[4] A. K. S. Kushwah and A. K. Manjhvar, *A Review on Link Prediction in Social Network,* Int. J. Grid Distrib. Comput. **9**, 43 (2016).

[5] G. Andreatta and L. Romeo, *Stochastic Shortest Paths with Recourse,* Networks **18**, 193 (1988).

[6] L. Fu and L. Rilett, *Expected Shortest paths in Dynamic and Stochastic Traffic Networks,* Transp. Res. Part B Methodol. **32**, 499 (1998).

[7] W. Zeng and R. L. Church, *Finding Shortest Paths on Real Road Networks: the Case for A*,* Int. J. Geogr. Inf. Sci. **23**, 531 (2009).

[8] Y. M. Nie and X. Wu, *Shortest Path Problem Considering on-time Arrival Probability,* Transp. Res. Part B Methodol. **43**, 597 (2009).

[9] F. B. Zhan and C. E. Noon, *Shortest Path Algorithms: An Evaluation Using Real Road Networks,* Transp. Sci. **32**, 65

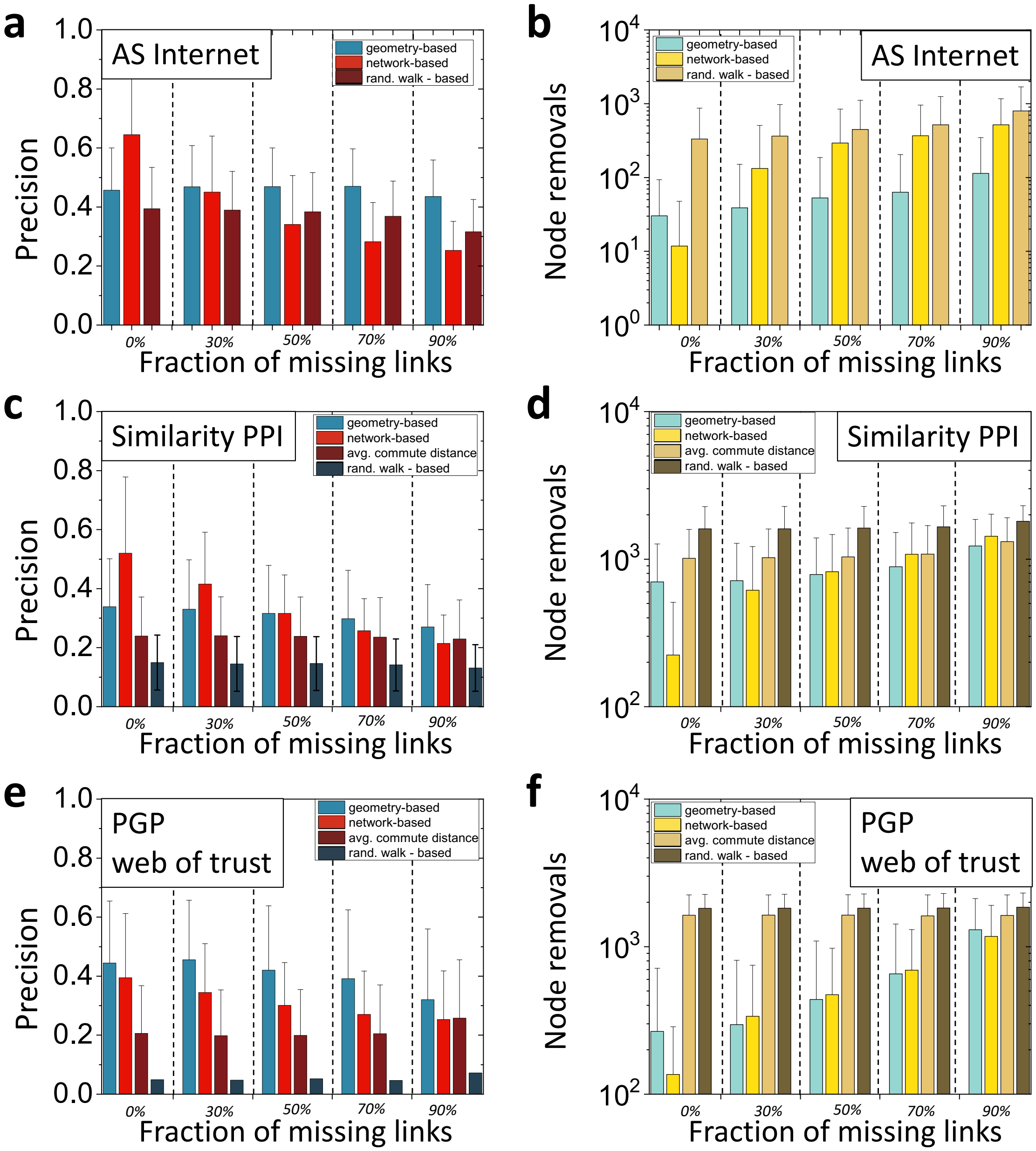

FIG. S14: The accuracy of path finding in incomplete real networks with present spurious links. The accuracy is quantified by (**a**,**c**,**e**) statistical precision and (**b**,**d**,**f**) the number of node removals to disconnect a node pair of interest. The networks are (**a**,**b**) the Internet, (**c**,**d**) similarity PPI, and (**e**,**f**) PGP web of trust.

(1998).

[10] B. Y. Chen, W. H. K. Lam, A. Sumalee, Q. Li, H. Shao, and Z. Fang, *Finding Reliable Shortest Paths in Road Networks Under Uncertainty,* Networks Spat. Econ. **13**, 123 (2013).

[11] A. Orda and R. Rom, *Shortest-Path and Minimum-Delay Algorithms in Networks with Time-Dependent Edge-Length,* J. ACM **37**, 607 (1990).

[12] G. Yu and J. Yang, *On the Robust Shortest Path Problem,* Comput. Oper. Res. **25**, 457 (1998).

[13] S. Peer and D. K. Sharma, *Finding the Shortest Path in Stochastic Networks,* Comput. Math. with Appl. **53**, 729 (2007).

[14] L. R. Nielsen, K. A. Andersen, and D. Pretolani, *Ranking Paths in Stochastic Time-Dependent Networks,* Eur. J. Oper. Res. **236**, 903 (2014).

[15] S. Kent, C. Lynn, and K. Seo, *Secure Border Gateway Protocol (S-BGP),* IEEE J. Sel. Areas Commun. **18**, 582 (2000).

[16] L. Subramanian, V. Roth, I. Stoica, S. Shenker, and R. H. Katz, in *1st Symp. Networked Syst. Des. Implementation, NSDI 2004* (USENIX Association, 2004).

[17] J. Karlin, S. Forrest, and J. Rexford, in *Proc. 2006 IEEE Int. Conf. Netw. Protoc.* (IEEE, 2006) pp. 290–299.

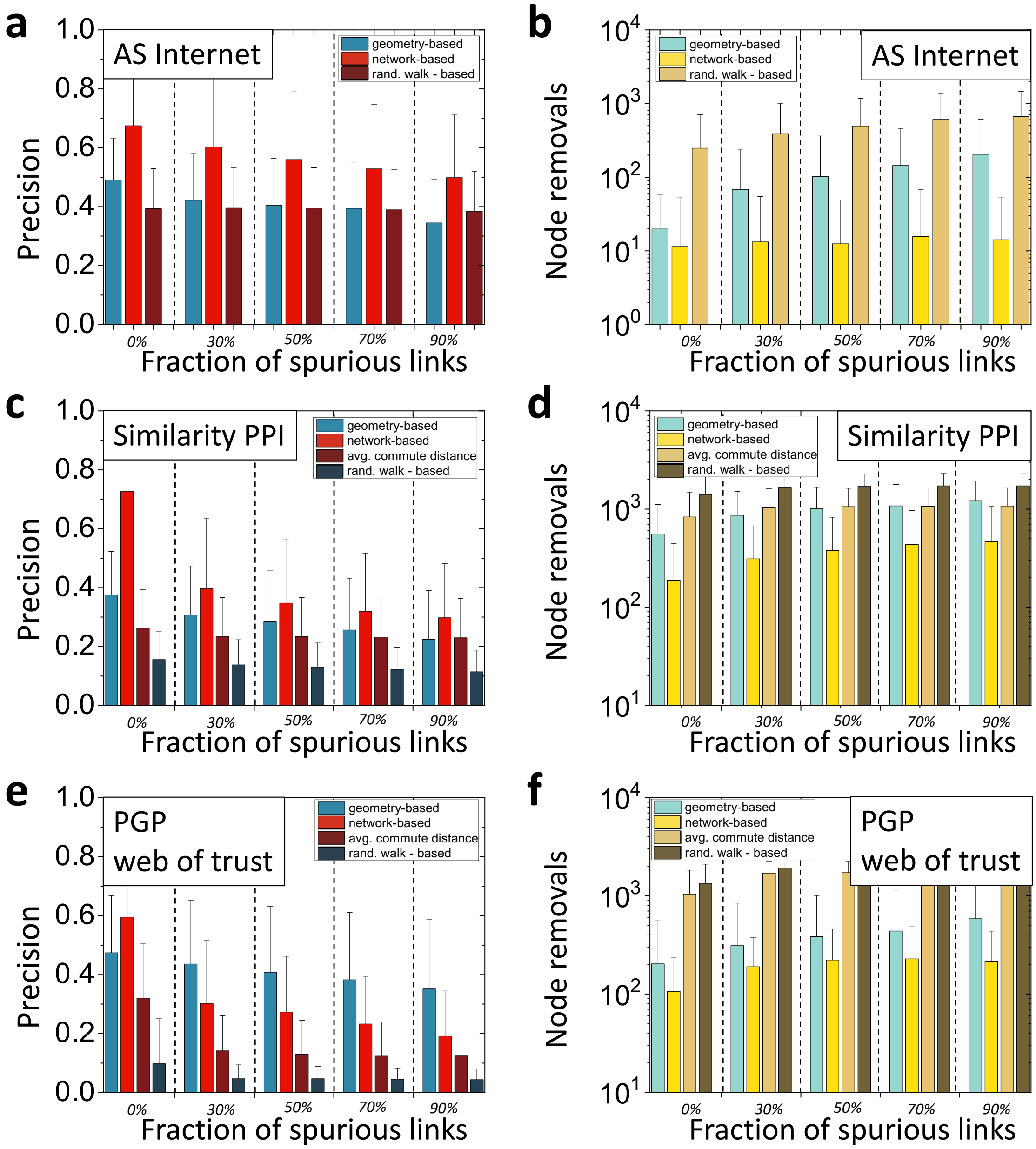

FIG. S15: The accuracy of path finding in real networks with spurious links. The accuracy is quantified by (**a,c,e**) statistical precision and (**b,d,f**) the number of node removals to disconnect a node pair of interest. The networks are (**a,b**) the Internet, (**c,d**) similarity PPI, and (**e,f**) PGP web of trust.

[18] M. Lepinski and S. Kent, *RFC 6480 - An Infrastructure to Support Secure Internet Routing,* IETF (2012).
[19] M. Lepinski and K. Sriram, *RFC-BGPsec Protocol Specification,* rfc-8205 (2017).
[20] M. Lad, D. Massey, D. Pei, Y. Wu, B. Zhang, and L. Zhang, in *15th USENIX Secur. Symp.* (2006).
[21] C. Zheng, L. Ji, D. Pei, J. Wang, and P. Francis, in *Proc. 2007 Conf. Appl. Technol. Archit. Protoc. Comput. Commun. - SIGCOMM '07* (2007) p. 277.
[22] X. Hu and Z. M. Mao, in *2007 IEEE Symp. Secur. Priv. (SP '07)* (IEEE, 2007) pp. 3–17.
[23] J. Schlamp, R. Holz, Q. Jacquemart, G. Carle, and E. W. Biersack, *HEAP: Reliable Assessment of BGP Hijacking Attacks,* IEEE J. Sel. Areas Commun. **34**, 1849 (2016).
[24] Y.-J. Chi, R. Oliveira, and L. Zhang, *Cyclops: the AS-level Connectivity Observatory,* ACM SIGCOMM Comput. Commun. Rev. **38**, 5 (2008).
[25] Z. Zhang, Y. Zhang, Y. C. Hu, Z. M. Mao, and R. Bush, *iSPY: Detecting IP Prefix Hijacking on My Own,* IEEE/ACM Trans. Netw. **18**, 1815 (2010).
[26] X. Shi, Y. Xiang, Z. Wang, X. Yin, and J. Wu, in *Proc. 2012 ACM Conf. Internet Meas. Conf. - IMC '12* (ACM Press,

2012) p. 15.

[27] P. Sermpezis, V. Kotronis, P. Gigis, X. Dimitropoulos, D. Cicalese, A. King, and A. Dainotti, *ARTEMIS: Neutralizing BGP Hijacking Within a Minute,* IEEE/ACM Trans. Netw. **26**, 2471 (2018).

[28] M. Kitsak, A. Elmokashfi, S. Havlin, and D. Krioukov, *Long-Range Correlations and Memory in the Dynamics of Internet Interdomain Routing,* PLoS One **10**, e0141481 (2015).

[29] T. Martin, G. Karopoulos, J. L. Hernández-Ramos, G. Kambourakis, and I. Nai Fovino, *Demystifying COVID-19 Digital Contact Tracing: A Survey on Frameworks and Mobile Apps,* Wirel. Commun. Mob. Comput. **2020**, 1 (2020).

[30] K. Luck, D.-K. Kim, L. Lambourne, K. Spirohn, B. E. Begg, W. Bian, R. Brignall, T. Cafarelli, F. J. Campos-Laborie, B. Charloteaux, D. Choi, A. G. Coté, M. Daley, S. Deimling, A. Desbuleux, A. Dricot, M. Gebbia, M. F. Hardy, N. Kishore, J. J. Knapp, I. A. Kovács, I. Lemmens, M. W. Mee, J. C. Mellor, C. Pollis, C. Pons, A. D. Richardson, S. Schlabach, B. Teeking, A. Yadav, M. Babor, D. Balcha, O. Basha, C. Bowman-Colin, S.-F. Chin, S. G. Choi, C. Colabella, G. Coppin, C. D'Amata, D. De Ridder, S. De Rouck, M. Duran-Frigola, H. Ennajdaoui, F. Goebels, L. Goehring, A. Gopal, G. Haddad, E. Hatchi, M. Helmy, Y. Jacob, Y. Kassa, S. Landini, R. Li, N. van Lieshout, A. MacWilliams, D. Markey, J. N. Paulson, S. Rangarajan, J. Rasla, A. Rayhan, T. Rolland, A. San-Miguel, Y. Shen, D. Sheykhkarimli, G. M. Sheynkman, E. Simonovsky, M. Taan, A. Tejeda, V. Tropepe, J.-C. Twizere, Y. Wang, R. J. Weatheritt, J. Weile, Y. Xia, X. Yang, E. Yeger-Lotem, Q. Zhong, P. Aloy, G. D. Bader, J. De Las Rivas, S. Gaudet, T. Hao, J. Rak, J. Tavernier, D. E. Hill, M. Vidal, F. P. Roth, and M. A. Calderwood, *A Reference Map of the Human Binary Protein Interactome,* Nature **580**, 402 (2020).

[31] J. Cannon, W. Floyd, R. Kenyon, and W. Parry, in *Flavors Geom.*, edited by S. Levy (MSRI, Berkeley, 1997) pp. 59–116.

[32] D. Krioukov, F. Papadopoulos, A. Vahdat, and M. Boguñá, *Curvature and Temperature of Complex Networks,* Phys. Rev. E **80**, 35101 (2009).

[33] D. Krioukov, F. Papadopoulos, M. Kitsak, A. Vahdat, and M. Boguñá, *Hyperbolic Geometry of Complex Networks,* Phys. Rev. E **82**, 036106 (2010).

[34] M. Boguñá, F. Papadopoulos, and D. Krioukov, *Sustaining the Internet with Hyperbolic Mapping.* Nat. Commun. **1**, 62 (2010).

[35] F. Papadopoulos, C. Psomas, and D. Krioukov, *Network Mapping by Replaying Hyperbolic Growth,* IEEE/ACM Trans. Netw. **23**, 198 (2015).

[36] F. Papadopoulos, R. Aldecoa, and D. Krioukov, *Network Geometry Inference Using Common Neighbors,* Phys. Rev. E **92**, 022807 (2015).

[37] M. Kitsak, F. Papadopoulos, and D. Krioukov, *Latent Geometry of Bipartite Networks,* Phys. Rev. E **95**, 032309 (2017).

[38] R. Aldecoa, C. Orsini, and D. Krioukov, *Hyperbolic Graph Generator,* Comput. Phys. Commun. **196**, 492 (2015).

[39] G. García-Pérez, M. Boguñá, and M. Á. Serrano, *Multiscale Unfolding of Real Networks by Geometric Renormalization,* Nat. Phys. **14**, 583 (2018).

[40] K. Zuev, M. Boguñá, G. Bianconi, and D. Krioukov, *Emergence of Soft Communities from Geometric Preferential Attachment,* Sci. Rep. **5**, 9421 (2015).

[41] A. Muscoloni and C. V. Cannistraci, *A Nonuniform Popularity-Similarity Optimization (nPSO) Model to Efficiently Generate Realistic Complex Networks with Communities,* New J. Phys. **20**, 052002 (2018).

[42] M. Boguñá and R. Pastor-Satorras, *Class of Correlated Random Networks with Hidden Variables,* Phys. Rev. E **68**, 036112 (2003).

[43] M. Gromov (Springer, New York, NY, 1987) pp. 75–263.

[44] A. Clauset, C. Moore, and M. E. J. Newman, *Hierarchical Structure and the Prediction of Missing Links in Networks,* Nature **453**, 98 (2008).

[45] M. Kitsak, I. Voitalov, and D. Krioukov, *Link Prediction with Hyperbolic Geometry,* Phys. Rev. Res. **2**, 043113 (2020).

[46] M. Faloutsos, P. Faloutsos, and C. Faloutsos, in *ACM SIGCOMM computer communication review*, Vol. 29 (ACM, 1999) pp. 251–262.

[47] L. Subramanian, S. Agarwal, J. Rexford, and R. H. Katz, in *Proceedings. Twenty-First Annual Joint Conference of the IEEE Computer and Communications Societies*, Vol. 2 (IEEE, 2002) pp. 618–627.

[48] Lixin Gao, *On Inferring Autonomous System Relationships in the Internet,* IEEE/ACM Trans. Netw. **9**, 733 (2001).

[49] M. Luckie, B. Huffaker, A. Dhamdhere, V. Giotsas, *et al.*, in *Proceedings of the 2013 conference on Internet measurement conference* (ACM, 2013) pp. 243–256.

[50] Y. Rekhter, T. Li, and S. Hares, *A Border Gateway Protocol 4 (BGP-4),* Internet Engineering Task Force, http://www.rfc-editor. org/rfc/rfc4271. txt, access on **6**, 34 (2014).

[51] *A hijack of financial services,* `https://arstechnica.com/security/2017/04/russian-controlled-telecom-hijacks-financial-ser` (2017).

[52] *Youtube hijack,* `https://www.ripe.net/publications/news/industry-developments/youtube-hijacking-a-ripe-ncc-ris-case-st` (2008).

[53] P.-A. Vervier, O. Thonnard, and M. Dacier, in *Proc. 2015 Netw. Distrib. Syst. Secur. Symp.* (Internet Society, Reston, VA, 2015).

[54] *Massive route leak causes Internet slowdown,* `https://bgpmon.net/massive-route-leak-cause-internet-slowdown/` (2015).

[55] *Internet vulnerability takes down Google,* `https://blog.thousandeyes.com/internet-vulnerability-takes-down-google/` (2018).

[56] P. Sermpezis, V. Kotronis, A. Dainotti, and X. Dimitropoulos, *A Survey among Network Operators on BGP Prefix*

Hijacking, ACM SIGCOMM Comput. Commun. Rev. **48**, 64 (2018).
[57] A. Dhamdhere and C. Dovrolis, *Twelve Years in the Evolution of the Internet Ecosystem,* IEEE/ACM Trans. Netw. **19**, 1420 (2011).
[58] J. Das and H. Yu, *HINT: High-Quality Protein Interactomes and their Applications in Understanding Human Disease,* BMC Syst. Biol. **6**, 92 (2012).
[59] A. J. McCoy, V. Chandana Epa, and P. M. Colman, *Electrostatic Complementarity at Protein/Protein Interfaces,* J. Mol. Biol. **268**, 570 (1997).
[60] B. A. Lewis and D. M. Engelman, *Lipid Bilayer Thickness Varies Linearly with Acyl Chain Length in Fluid Phosphatidylcholine Vesicles,* J. Mol. Biol. **166**, 211 (1983).
[61] Y. Yano and K. Matsuzaki, *Measurement of Thermodynamic Parameters for Hydrophobic Mismatch 1: Self-Association of a Transmembrane Helix ,* Biochemistry **45**, 3370 (2006).
[62] A. V. Botelho, T. Huber, T. P. Sakmar, and M. F. Brown, *Curvature and Hydrophobic Forces Drive Oligomerization and Modulate Activity of Rhodopsin in Membranes,* Biophys. J. **91**, 4464 (2006).
[63] Q. Zhang, M. Sanner, and A. J. Olson, *Shape Complementarity of Protein-Protein Complexes at Multiple Resolutions,* Proteins Struct. Funct. Bioinforma. **75**, 453 (2009).
[64] Y. Li, X. Zhang, and D. Cao, *The Role of Shape Complementarity in the Protein-Protein Interactions,* Sci. Rep. **3**, 3271 (2013).
[65] M. A. Serrano, M. Boguna, and A. Vespignani, *Extracting the Multiscale Backbone ofComplex Weighted Networks,* Proc. Natl. Acad. Sci. **106**, 6483 (2009).
[66] M. Kanehisa, M. Furumichi, M. Tanabe, Y. Sato, and K. Morishima, *KEGG: New Perspectives on Genomes, Pathways, Diseases and Drugs,* Nucleic Acids Res. **45**, D353 (2017).
[67] L. K. Teixeira and S. I. Reed, *Ubiquitin Ligases and Cell Cycle Control,* Annu. Rev. Biochem. **82**, 387 (2013).
[68] R. Derynck and Y. E. Zhang, *Smad-Dependent and Smad-Independent Pathways in TGF-β Family Signalling,* Nature **425**, 577 (2003).
[69] A. A. Eisa, S. De, A. Detwiler, E. Gilker, A. C. Ignatious, S. Vijayaraghavan, and D. Kline, *YWHA (14-3-3) Protein Isoforms and their Interactions with CDC25B Phosphatase in Mouse Oogenesis and Oocyte Maturation,* BMC Dev. Biol. **19**, 20 (2019).
[70] D. W. Huang, B. T. Sherman, and R. A. Lempicki, *Systematic and Integrative Analysis of Large Gene Lists using DAVID Bioinformatics Resources,* Nat. Protoc. **4**, 44 (2009).
[71] H. Hu and S.-C. Sun, *Ubiquitin Signaling in Immune Responses,* Cell Res. **26**, 457 (2016).
[72] L. Deng, T. Meng, L. Chen, W. Wei, and P. Wang, *The Role of Ubiquitination in Tumorigenesis and Targeted Drug Discovery,* Signal Transduct. Target. Ther. **5**, 11 (2020).
[73] J. K. Gustin, A. V. Moses, K. Früh, and J. L. Douglas, *Viral Takeover of the Host Ubiquitin System,* Front. Microbiol. **2**, 161 (2011).
[74] L. Geng, C. J. Huntoon, and L. M. Karnitz, *RAD18-Mediated Ubiquitination of PCNA Activates the Fanconi Anemia DNA Repair Network,* J. Cell Biol. **191**, 249 (2010).
[75] J. K. Morrow, H.-K. Lin, S.-C. Sun, and S. Zhang, *Targeting Ubiquitination for Cancer Therapies,* Future Med. Chem. **7**, 2333 (2015).
[76] M. Bienko, *Ubiquitin-Binding Domains in Y-Family Polymerases Regulate Translesion Synthesis,* Science **310**, 1821 (2005).
[77] A. Peisley, B. Wu, H. Xu, Z. J. Chen, and S. Hur, *Structural Basis for Ubiquitin-Mediated Antiviral Signal Activation by RIG-I,* Nature **509**, 110 (2014).
[78] A. M. Ali, A. Pradhan, T. R. Singh, C. Du, J. Li, K. Wahengbam, E. Grassman, A. D. Auerbach, Q. Pang, and A. R. Meetei, *FAAP20: a Novel Ubiquitin-Binding FA Nuclear Core-Complex Protein Required for Functional Integrity of the FA-BRCA DNA Repair Pathway,* Blood **119**, 3285 (2012).
[79] S. Rahighi, F. Ikeda, M. Kawasaki, M. Akutsu, N. Suzuki, R. Kato, T. Kensche, T. Uejima, S. Bloor, D. Komander, F. Randow, S. Wakatsuki, and I. Dikic, *Specific Recognition of Linear Ubiquitin Chains by NEMO Is Important for NF-κB Activation,* Cell **136**, 1098 (2009).
[80] *The Open PGP Alliance,* `http://www.openpgp.org/`.
[81] *OpenPGP web of trust database,* `http://www.lysator.liu.se/~jc/wotsap/wots2/`.
[82] F. Göbel and A. Jagers, *Random walks on graphs,* Stoch. Process. their Appl. **2**, 311 (1974).
[83] F. Fouss, A. Pirotte, J. M. Renders, and M. Saerens, *Random-walk computation of similarities between nodes of a graph with application to collaborative recommendation,* IEEE Trans. Knowl. Data Eng. **19**, 355 (2007).
[84] R. Penrose, *A generalized inverse for matrices,* Math. Proc. Cambridge Philos. Soc. **51**, 406 (1955).
