# Supplementary material for: Finding shortest and nearly shortest path nodes in large substantially incomplete networks by hyperbolic mapping
Source: Nat Commun. 2023 Jan 17;14:186. doi: 10.1038/s41467-022-35181-w (PMC9845360; doi:10.1038/s41467-022-35181-w)
Supplement: Supplementary file 3 — Description of additional Supplementary File [file 41467_2022_35181_MOESM3_ESM.pdf]

<u>Descriptions of additional Supplementary Files</u>

Supplementary_Data1.zip file contains Internet networks, their hyperbolic coordinates, and the routing path data.

Supplementary_Data2.zip file contains protein-protein interaction networks, their hyperbolic coordinates, and the cellular pathway data.
